# AMPK-related protein kinase ARK5 regulates subcellular localization of RNA-binding protein hnRNP A1 during hypertonic stress

**DOI:** 10.1016/j.jbc.2022.102364

**Published:** 2022-08-11

**Authors:** Krishna Bhattarai, Travis Richard, Thet Fatica, Brianna Frangione, William G. Willmore, Martin Holcik

**Affiliations:** 1 Department of Health Sciences, Carleton University, Ottawa, Ontario, Canada; 2 Department of Biochemistry, Microbiology, and Immunology, University of Ottawa, Ottawa, Ontario, Canada; 3 Department of Biology, Carleton University, Ottawa, Ontario, Canada

**Keywords:** hypertonic stress, heterogeneous nuclear ribonucleoprotein A1, AMPK-related protein kinase 5, phosphorylation, Bcl-xL, caspase, coimmunoprecipitation, DMSO, dimethyl sulfoxide, IP, immunoprecipitation, KD, kinase-dead, P/S, penicillin/streptomycin, PVDF, polyvinylidene difluoride, TBS, Tris-buffered saline

## Abstract

The heterogeneous nuclear ribonucleoprotein hnRNP A1 is a nucleocytoplasmic-shuttling RNA-binding protein that plays an important role in nucleic acid metabolism and gene expression regulation. The function of hnRNP A1 is determined in part by its specific location within the cell. Although some work has been done to elucidate the signaling pathways that regulate the cellular localization of hnRNP A1, the precise mechanism(s), including physiological and pathophysiological conditions that alter hnRNP A1 localization, are not known. We previously conducted an unbiased RNAi-based kinome-wide screen to identify kinases that regulate hnRNP A1 localization during hypertonic stress. One of the hits from this screen is AMPK-related protein kinase 5 (ARK5). Here, we validate ARK5 as the kinase responsible for controlling hnRNP A1 subcellular localization in response to hypertonic stress. We find using immunoprecipitation and *in vitro* kinase assay methods that ARK5 directly interacts with and phosphorylates hnRNP A1 on serine residues within the F-peptide region. We further show that the M9 motif of hnRNP A1 is essential for the ARK5-hnRNP A1 interaction and subsequent phosphorylation. In addition, the silencing of ARK5 increases the expression of antiapoptotic protein Bcl-xL and consequently delays caspase activation during hypertonic stress. Our results indicate that ARK5 phosphorylates hnRNP A1 and regulates its subcellular localization during hypertonic stress.

Protein synthesis, or mRNA translation, has been recognized as a critical checkpoint for the maintenance of cellular homeostasis and the cellular stress response (1). Furthermore, regulation of protein synthesis provides cells with the ability to adjust their protein composition and levels rapidly and specifically in response to different forms of cellular stresses (2). The diverse specialized RNA-binding proteins (RBPs) play a major role in protein synthesis by acting on an mRNA and forming ribonucleoprotein (RNP) complexes (3, 4, 5). Among the RBPs, the heterogeneous nuclear ribonucleoproteins (hnRNPs) are among the most abundant and well-studied families of RBPs; within this family, the prototypical protein is the heterogeneous nuclear ribonucleoprotein A1 (hnRNP A1). hnRNP A1 fulfills multiple functions in cellular nucleic acid metabolism and regulation of gene expression including transcriptional regulation, alternative splicing, mRNA stabilization, maturation of nascent transcripts, polyadenylation, nuclear mRNA export, and translational control (6).

hnRNP A1 binds RNA through two RNA recognition motifs in its N-terminal domain (1–196 aa) and several RGG repeats in its C-terminal domain (197–320 aa). It harbors an M9 motif (268–305 aa) at its C terminus, which acts as a nuclear localization signal and is involved in hnRNP A1 nuclear import and export (7, 8, 9). hnRNP A1 shuttles between the nucleus and the cytoplasm, with the bulk of the protein displaying nuclear localization under normal growth conditions (10). However, hnRNP A1 undergoes posttranslational modification in response to cellular stresses, such as during hypertonic stress; several serine residues between 301 and 318 aa segment (termed “F-peptide”) are phosphorylated, which results in a conformational change of the M9 motif (11). This, in turn, blocks hnRNP A1 interaction with transportin-1/2 and consequently prevents its nuclear import (11). In addition to the structural stabilization of the M9 motif, hnRNP A1 transport also requires an intact M9 motif for both import and export of the protein (12, 13, 14). Previous studies identified several kinases involved in the shuttling of hnRNP A1 during stress. For example, it was shown that hexokinase 2 (HK2) and p38 mitogen-activated protein kinase (MAPK) are responsible for cytoplasmic accumulation of hnRNP A1 in response to hypertonic stress (15, 16). In addition, hnRNP A1 is also a substrate of S6K2 downstream of fibroblast growth factor-2 (FGF-2) signaling whereby S6K2 phosphorylates hnRNP A1 on serines 4 and 6, sites distinct from the F-peptide, resulting in an increase of the cytoplasmic hnRNP A1 levels (17). Furthermore, the accumulation of hnRNP A1 in the cytoplasm appears to have different consequences for distinct mRNAs. The cytoplasmic hnRNP A1 destabilizes mRNA of cIAP1 in UV-irradiated cells (18), suppresses internal ribosome entry site (IRES)–mediated translation of X chromosome-linked IAP (XIAP) and B-cell lymphoma-extra large (Bcl-xL) during hypertonic shock (19), promotes nuclear export of XIAP and Bcl-xL mRNAs in FGF-2 stimulated cells (17), and drives translation of human rhinovirus RNA for efficient infection (20).

Overall, these observations suggest that there exist multiple signaling pathways that control hnRNP A1 localization and their activation is stress specific. However, the kinases, precise molecular mechanisms controlling hnRNP A1 accumulation in distinct cellular compartments, and the biological consequences of distinct hnRNP A1 localization all remain unclear. In our previous work, we set out to determine the signaling molecules that regulate the localization of hnRNP A1 and the biological consequences of this regulation during hypertonic stress. We screened a library of siRNA pools targeting the kinome subset of the human genome and identified several candidate kinases that regulate the cytoplasmic accumulation of hnRNP A1 (15). In this work, we demonstrate that AMPK-related kinase, ARK5 (aka NUAK1), directly interacts with hnRNP A1 *via* the M9 motif of hnRNP A1 in the nucleus and subsequently phosphorylates hnRNP A1 within the F-peptide. This phosphorylation controls the subcellular localization of hnRNP A1 and, consequently, cell survival during hypertonic stress.

## Results

### ARK5 controls subcellular localization of hnRNP A1 during hypertonic stress

**Figure 1:**
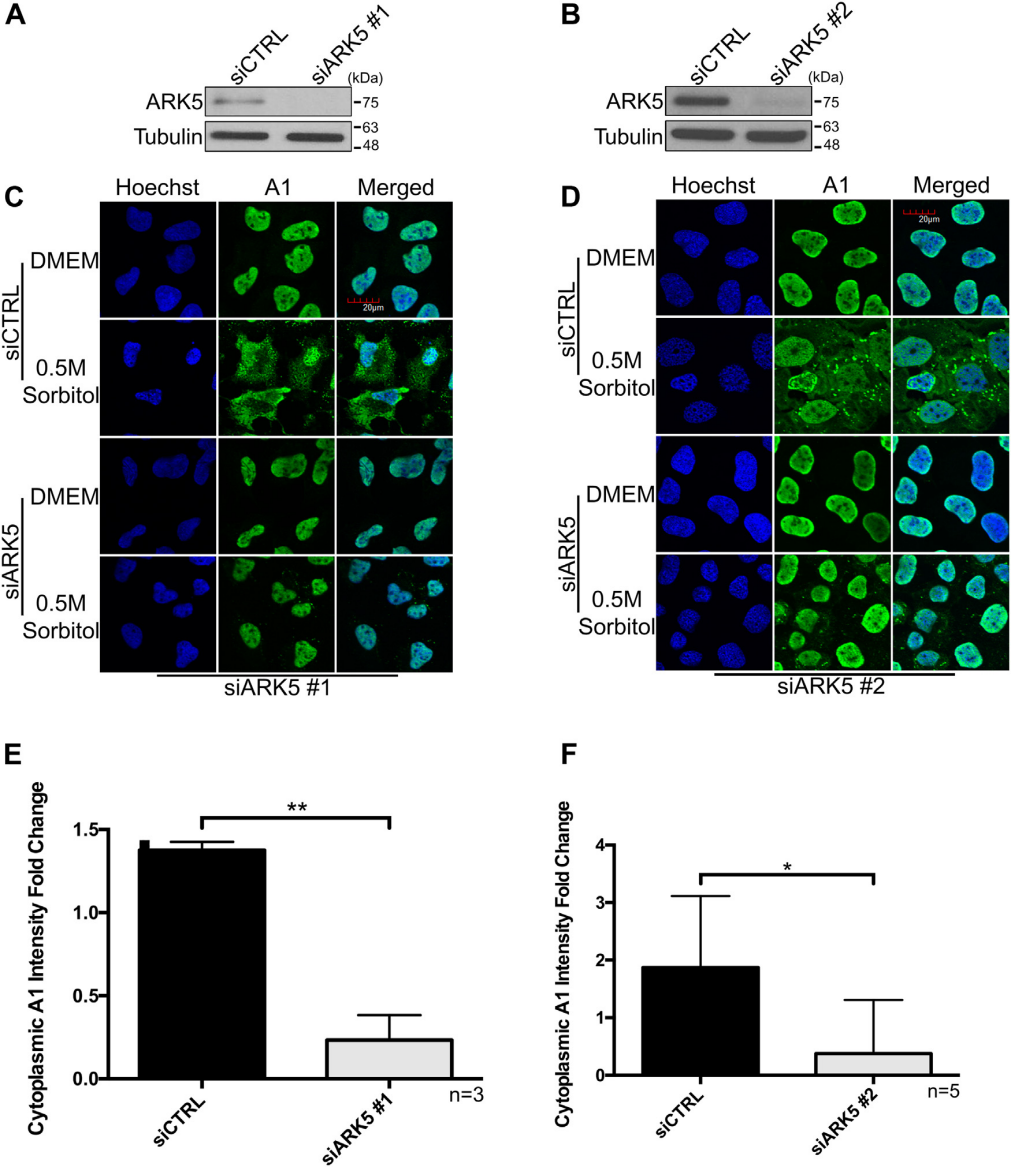
**Cytoplasmic accumulation of hnRNP A1 is dependent on ARK5 during hypertonic shock.** U2OS cells were transfected using two different, independent siRNAs for ARK5 to confirm the results of the kinome-wide screen. *A* and *B*, depict representative immunoblots of lysates from U2OS cells that had been transfected with either siARK5 (#1 & #2) or a negative control siRNA (siCTRL) under normal physiological conditions. Tubulin was used as a loading control. *C* and *D*, are representative immunofluorescence confocal microscopy images of U2OS cells treated for 2 h with DMEM or a 0.5 M sorbitol solution subsequent to the transfection with siRNA. Nuclei were stained with Hoechst stain. Images were taken using the 60×, water-based objective. Scale bar shown in the *top right image* pertains to all images. *E* and *F*, fold change quantification of cytoplasmic hnRNP A1 intensity for DMEM *versus* hypertonic shock treated cells of replicates of (*C*) and (*D*), respectively (∗*p* < 0.05; ∗∗*p* < 0.01). The images were analyzed on a Columbus Image Analysis Server (PerkinElmer) using an embedded Acapella Image Analysis Software (PerkinElmer) script as previously described (15).

**Figure 2:**
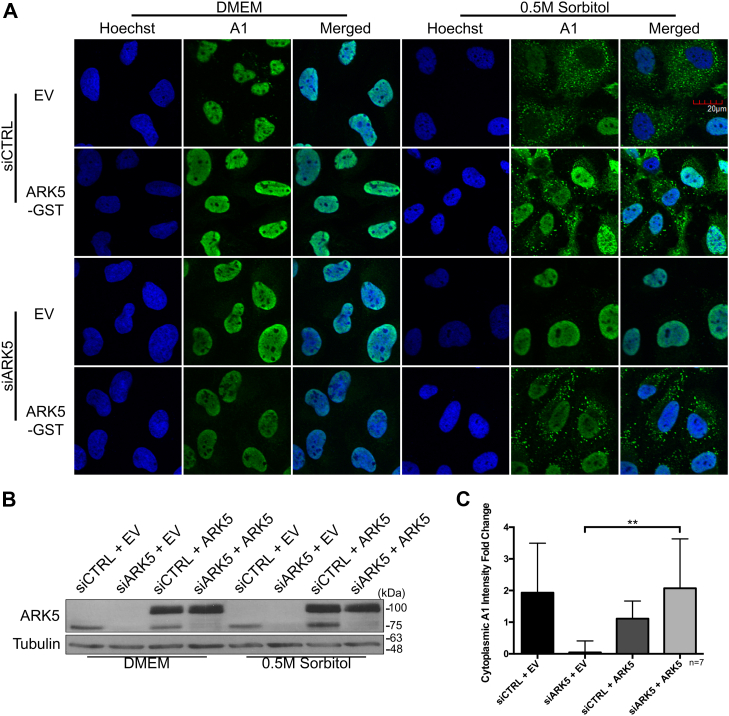
**Cytoplasmic accumulation of hnRNP A1 is restored by exogenous ARK5 expression.** U2OS cells were transfected with a negative control siRNA (siCTRL) or siARK5, followed by transfection of a negative control plasmid (EV, empty vector) or a GST-tagged ARK5 plasmid (ARK5-GST or ARK5) and subsequently treated with DMEM or 0.5 M sorbitol. *A*, representative immunofluorescence confocal microscopy images of U2OS cells. Nuclei were stained with Hoechst stain. Scale bar shown in the top right image pertains to all images. *B*, immunoblot confirmation of ARK5 knockdown and overexpression. *C*, fold change quantification of cytoplasmic hnRNP A1 intensity for DMEM *versus* hypertonic shock treated cells of replicates of (*A*) (∗∗*p* < 0.01). The images were analyzed on a Columbus Image Analysis Server (PerkinElmer) using an embedded Acapella Image Analysis Software (PerkinElmer) script as previously described (15).

**Figure 3:**
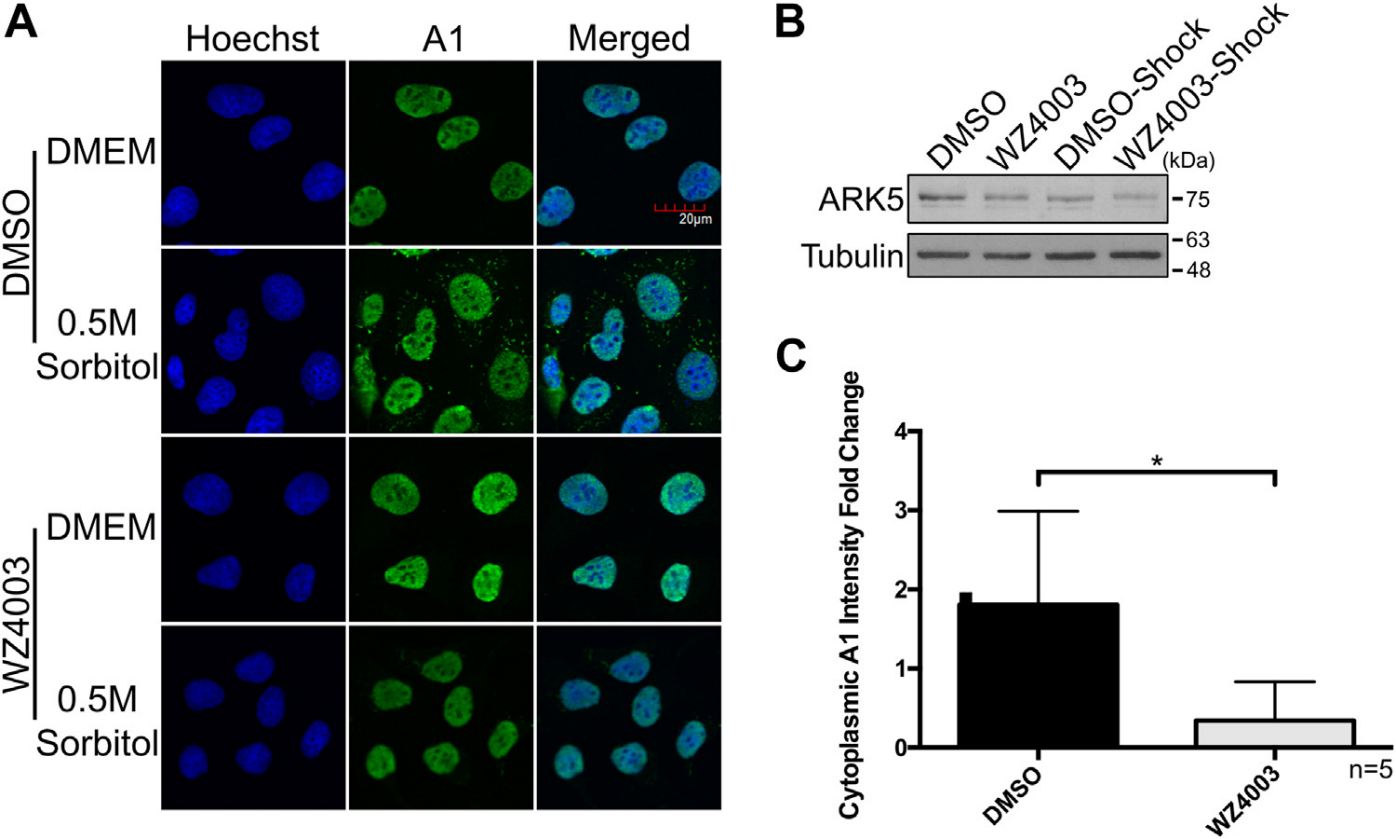
**ARK5 inhibitor, WZ4003, inhibits cytoplasmic accumulation of hnRNP A1 during hypertonic stress.** U2OS cells were pretreated with either DMSO or WZ4003, followed by a combination treatment of DMEM or 0.5 M sorbitol including either DMSO or WZ4003. *A*, representative immunofluorescence confocal microscopy images of treated U2OS cells. Nuclei were stained with Hoechst. Scale bar shown in the *top right image* pertains to all images. *B*, U2OS cells lysates were subjected to immunoblotting with the indicated antibodies. *C*, fold change quantification of cytoplasmic hnRNP A1 intensity for DMEM *versus* hypertonic shock treated cells of replicates of (*A*) (∗*p* < 0.05). The images were analyzed on a Columbus Image Analysis Server (PerkinElmer) using an embedded Acapella Image Analysis Software (PerkinElmer) script as previously described (15). DMSO, dimethyl sulfoxide.

**Supplemental Figure S1:**
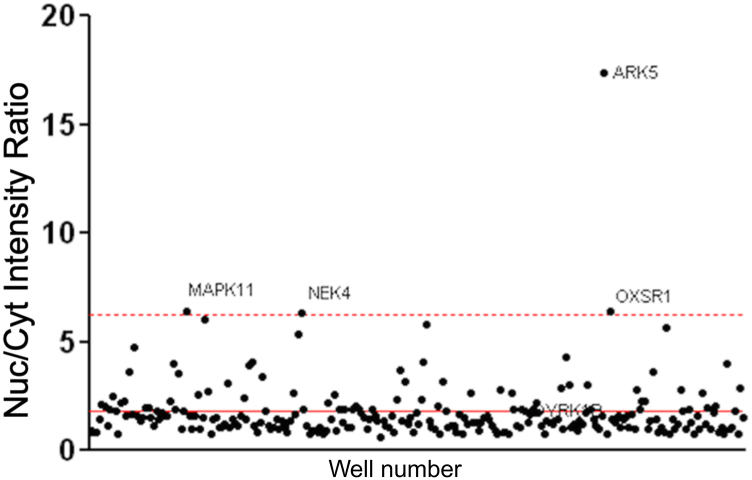
**Results of previously published RNAi-based kinome-wide screen identify ARK5 as a potential kinase which controls hnRNP A1 shuttling between nucleus and cytoplasm in response to hypertonic stress** (15)**.** U2OS cells were reverse transfected using an arrayed library of siRNA pools designated to target 691 genes related to the human kinome for a period of 72 h, followed by the induction of hypertonic stress *via* 0.6 M sorbitol for 4 h. The intensity ratios were calculated using immunofluorescence images depicting the nucleus *versus* cytoplasmic intensity ratio of hnRNP A1 localization during the screen as determined by the analysis of the Z-factor and plotted. *Solid red line*: mean of the plate; *Red dashed line*: 3σ. Only the results of one 384-well plate representing 1/3 of the screen are shown to aid in the visualization of ARK5 hit. Complete data is available in (15).

**Supplemental Figure S2:**
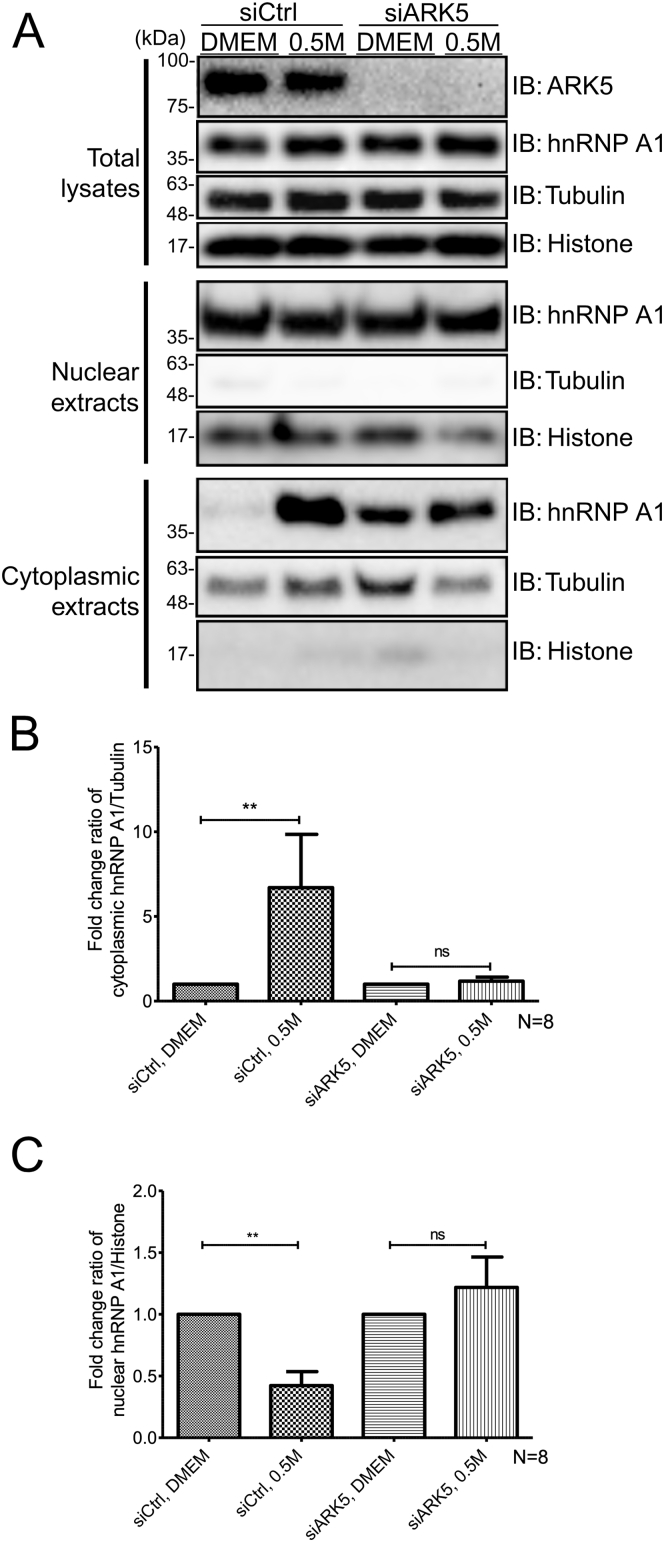
**Cytoplasmic accumulation of hnRNP A1 is dependent on ARK5 during hypertonic shock.** U2OS cells were transfected using siCTRL or siARK5 for 48 h and further treated with DMEM or 0.5 M sorbitol (0.5 M) for 2 h. *A*, immunoblots of subcellular fractionation of U2OS cell extracts. Tubulin and histone were used as loading controls. *B* and *C*, fold change ratio of cytoplasmic and nuclear hnRNP A1 relative to tubulin and histone, respectively, for their respective siRNA treatment. Band density was determined using ImageLab. (∗∗*p* < 0.01; data was analysed using one-tailed Wilcoxon signed-rank test).

We had previously performed an unbiased RNAi-based kinome-wide screen using an arrayed library designed to target 691 human kinome-related genes to determine the possible kinases that are responsible for the accumulation of hnRNP A1 in the cytoplasm during hypertonic stress and validated HK2 as a novel kinase to regulate the cytoplasmic accumulation of hnRNP A1 during hypertonic stress and rhinovirus infection (15). Here, we have selected one of the hits of this screen, ARK5 (aka NUAK1, UniProt ID O60285), for further validation and characterization (Fig. S1). First, we used two independent ARK5-targeting siRNAs (siARK5), which were different from the original siRNA pool used in the RNAi-based kinome screen, to confirm the screen results. As shown in Figure 1, *A*–*F*, both variants of siARK5s (#1 and #2) caused an approximate 5 to 6 fold change reduction in the cytoplasmic accumulation of hnRNP A1 when compared to the nontargeting control siRNA (siCTRL). As a further confirmation of the immunofluorescence data, we examined the subcellular distribution of hnRNP A1 by subcellular fractionation (Fig. S2). The siCtrl-treated cytoplasmic and nuclear extracts showed a significant increase and a decrease, respectively, of hnRNP A1 in sorbitol-treated cells when compared to Dulbecco's modified Eagle's medium (DMEM). In contrast, the siARK5-treated cytoplasmic and nuclear extracts did not show any significant changes in hnRNP A1 in sorbitol-treated cells when compared to DMEM. Since there is no change in total levels of hnRNP A1 in siARK5-treated cells, these data suggest that inhibiting ARK5 does not lead to the degradation of hnRNP A1 but instead prevents its cytoplasmic accumulation. To further confirm the dependence of hnRNP A1 localization on ARK5 during hypertonic stress, an ARK5 rescue experiment was performed. Importantly, the rescue of ARK5 expression in the siARK5-treated cells has shown a clear return to cytoplasmic accumulation of hnRNP A1 in hypertonic stressed cells (Fig. 2, *A*–*C*). In addition, to further demonstrate that cytoplasmic accumulation of hnRNP A1 in hypertonic stress cells is dependent on the activity of ARK5, we used WZ4003 compound, a high specificity inhibitor of ARK5 (21). Pretreatment of U2OS cells with WZ4003 before the hypertonic stress prevented the cytoplasmic accumulation of hnRNP A1, similarly to the levels seen in siARK5-treated cells (Fig. 3, *A*–*C*). Altogether, these results demonstrate that ARK5 is a *bona fide* regulator for cytoplasmic accumulation of hnRNP A1 in response to hypertonic stress.

### Silencing of ARK5 increases expression of Bcl-xL and represses caspase activation in stressed U2OS cells

**Figure 4:**
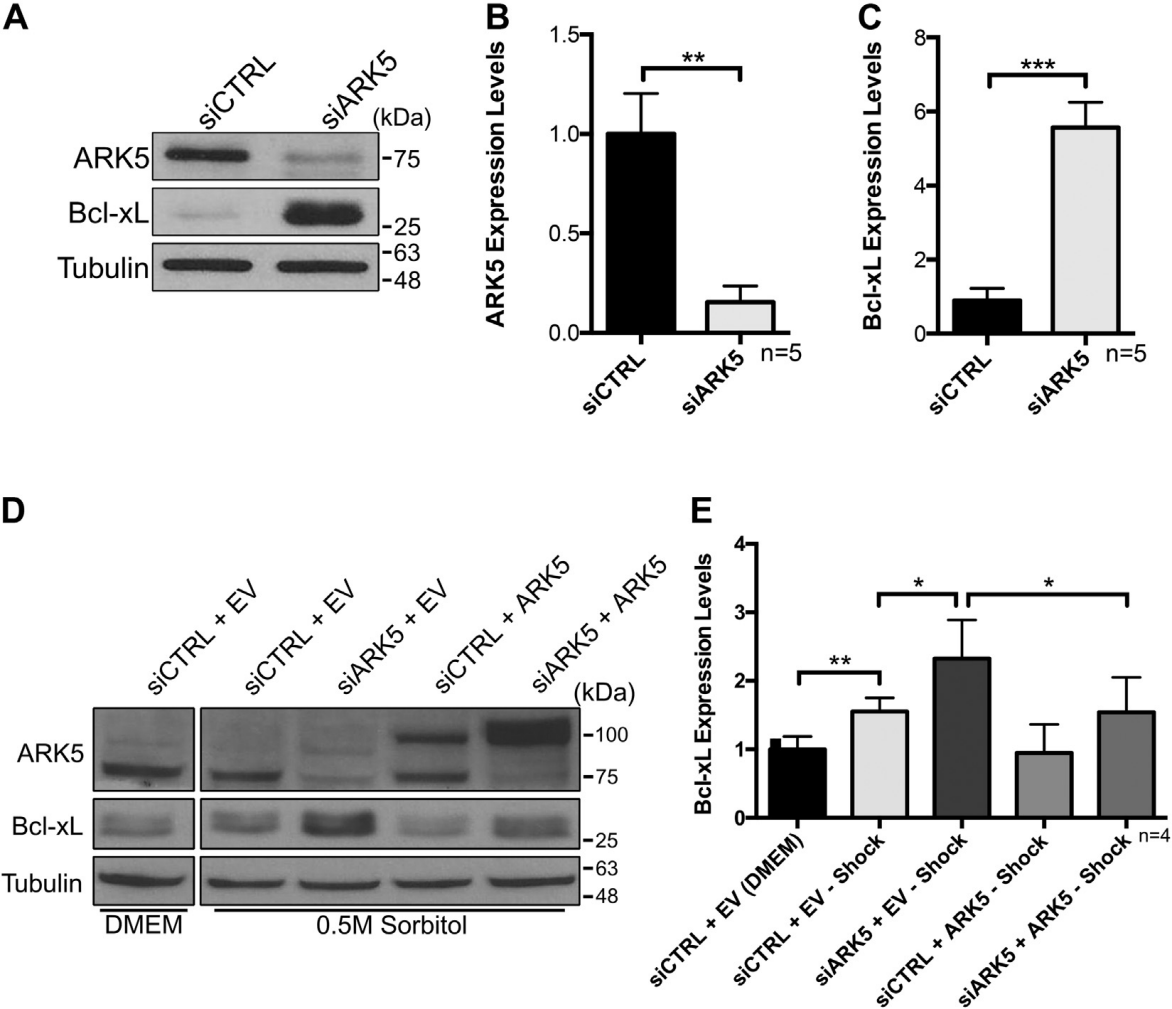
**Expression of Bcl-xL, a known target of hnRNP A1, is controlled by ARK5 during hypertonic stress.** U2OS cells were transfected with siCTRL (negative control) or siARK5 followed by transfection of a negative control plasmid (EV, empty vector) or a GST-tagged ARK5 plasmid (ARK5) as indicated. *A*–*C*, representative immunoblot and densitometry from repeated experiments of ARK5 and Bcl-xL levels of cells grown in DMEM and transfected with siRNA. *D* and *E*, representative immunoblot and densitometry data from repeated experiments of cells grown in 0.5 M sorbitol during an ARK5 expression rescue (siCTRL + EV (DMEM)) was set as 1. Tubulin was used as a loading control (∗*p* < 0.05; ∗∗*p* < 0.01).

hnRNP A1 has been identified as an IRES *trans*-acting factor (ITAF) for XIAP, Apaf-1, HRV, c-*myc*, and Bcl-xL (19, 20, 22). Therefore, we wished to examine the impact of ARK5 inhibition on Bcl-xL expression in the hypertonic stressed cells. U2OS cells were transfected using siCTRL (negative control) or siARK5 and followed by a transfection of a control plasmid (empty vector) or a GST-tagged ARK5 plasmid (ARK5). Our results show that siRNA-mediated knockdown of ARK5 leads to the concomitant increase of Bcl-xL (Fig. 4*C*), suggesting that ARK5 acts as a negative regulator of Bcl-xL. As described previously (19), we also noted a modest increase in Bcl-xL protein expression in hypertonic cells (Fig. 4*D*). Importantly, Bcl-xL expression in hypertonic stressed cells was further increased by 78% when also treated with siARK5, an increase which was blunted by exogenous ARK5 expression (Fig. 4*D*). These data demonstrate a link between the ARK5 and the expression of Bcl-xL.

**Figure 5:**
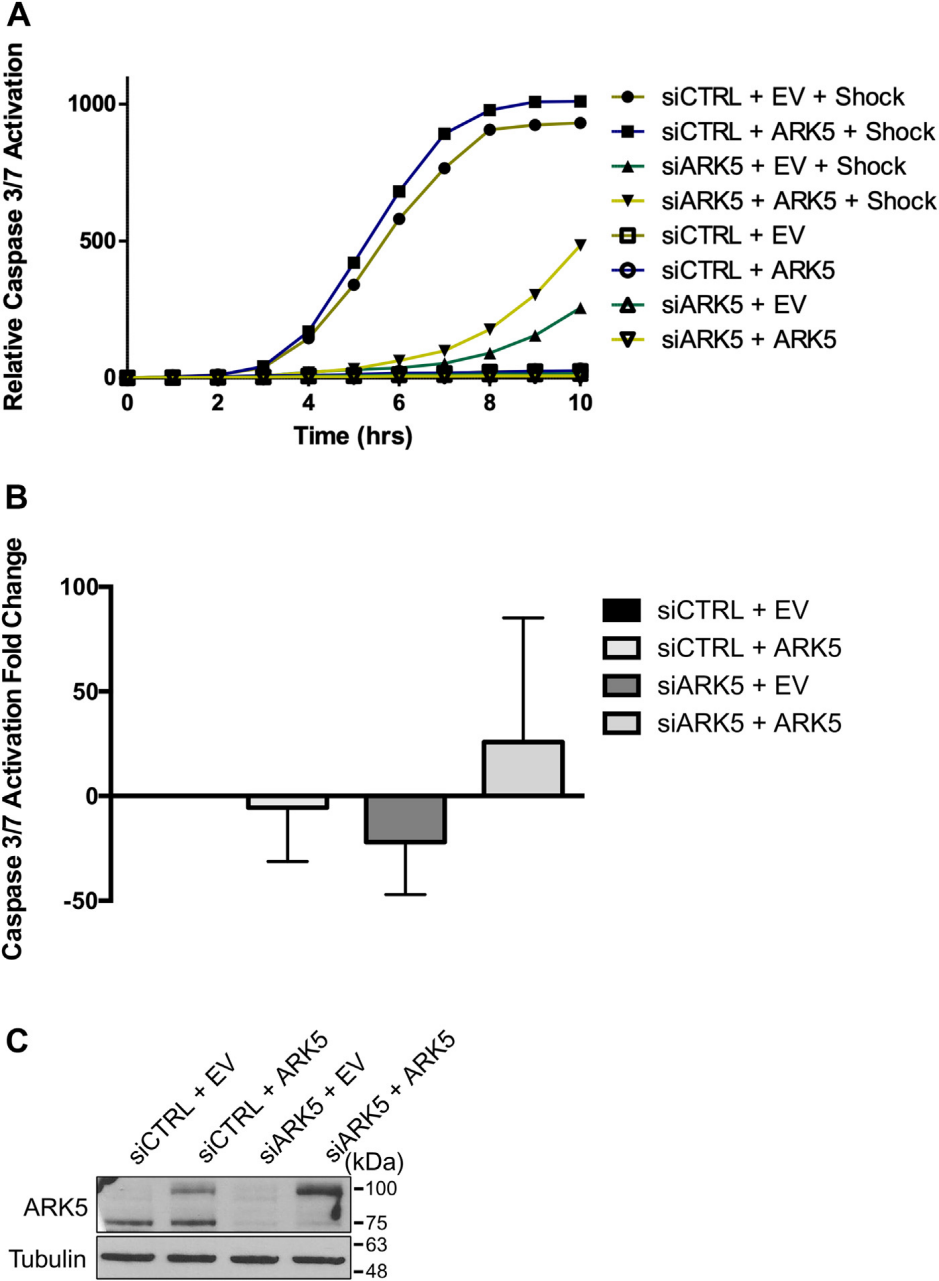
**Knockdown of ARK5 results in decreased caspase activation.** U2OS cells were transiently transfected with siCTRL (negative control) or siARK5 and followed by a negative control plasmid (EV, empty vector) or GST-tagged ARK5 plasmid (ARK5), subsequently treated with 0.5 M sorbitol and caspase 3/7 activity was monitored using IncuCyte live imaging platform. *A*, quantification of caspase 3 and 7 activation. The *y*-axis depicts the sum of fluorescent cells normalized to the percentage of cell confluency. Hollow shapes portray data of samples cultured in DMEM and solid shaped those maintained in 0.5 M sorbitol during the 10 h monitored. *B*, fold change caspase activation comparison between siCTRL-EV and the other treatment conditions at the 10 h time point. About 16 images were taken from each well and quantified. *C*, representative immunoblot from (*A*) using samples gathered in parallel. Tubulin was used as a loading control.

In response to stress, the regulation of IRES-mediated translation is believed to aid in the recovery of cells through the balanced synthesis or inhibition of proapoptotic or antiapoptotic proteins. However, exposure to extreme hypertonic stress results in inhibition of global protein synthesis, including the expression of proteins that serve to prevent apoptosis (19). As our results showed that the expression of Bcl-xL, an antiapoptotic protein, is altered by ARK5, we monitored the activation of caspases 3 and 7 to identify the impact on cell viability. Activation of caspases 3 and 7 over a 10 h period was measured using IncuCyte live imaging platform. Indeed, cells transiently transfected with siARK5 showed a marked delay in caspases 3/7 activation (Fig. 5*A*), as well as decreased levels of caspase activation (Fig. 5*B*), both of which were partially restored by exogenous ARK5 expression. The observed changes in caspase activation were congruent with the expression levels of Bcl-xL (Fig. 4*D*).

### The M9 motif and F-peptide regions of hnRNP A1 are necessary for ARK5 phosphorylation

Since we have confirmed the link between ARK5 and hnRNP A1’s subcellular localization, the next logical question raised are about the mechanism(s) by which ARK5 controls hnRNP A1. Does ARK5 control hnRNP A1 by direct phosphorylation or does it indirectly control it *via* another signaling pathway?

**Figure 6:**
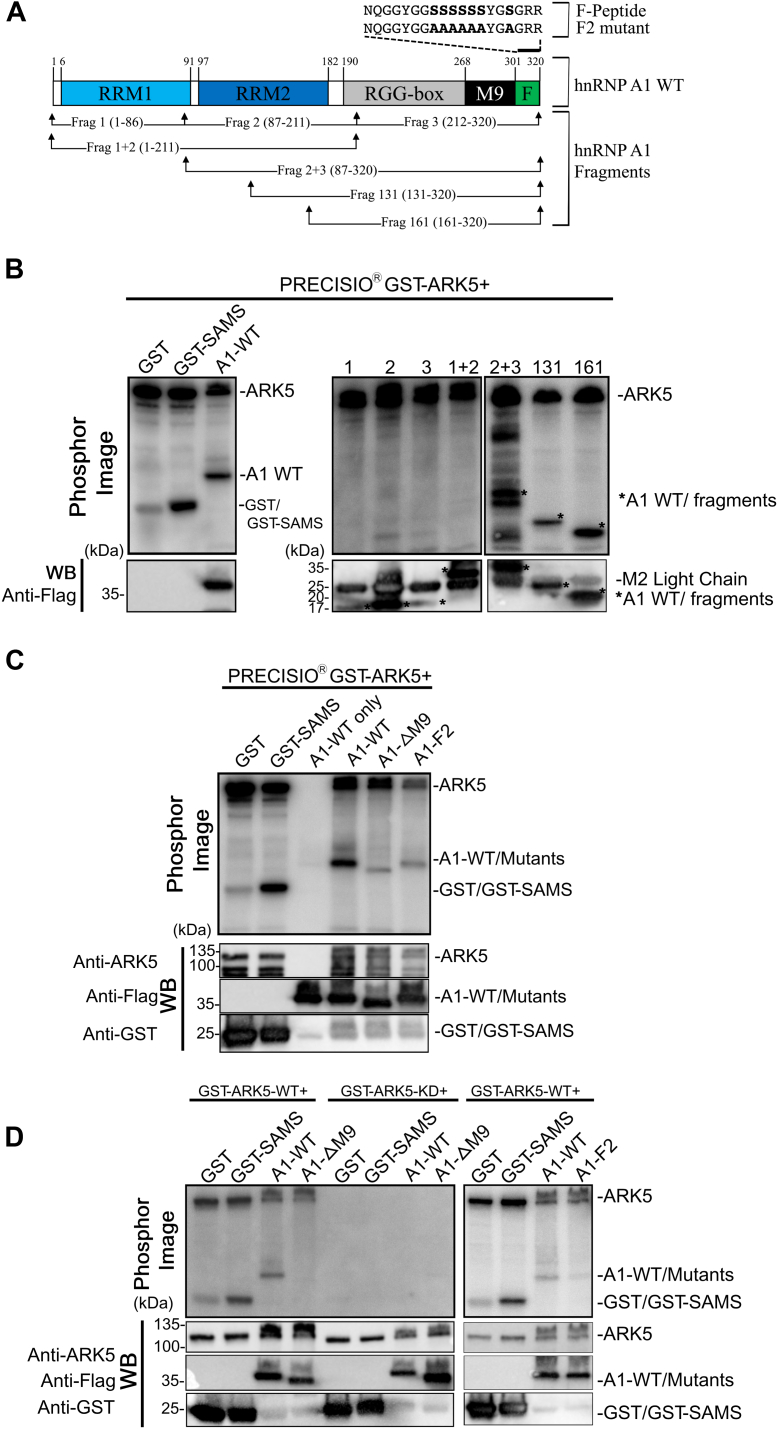
**The M9 motif and F-peptide regions of hnRNP A1 can impact phosphorylation efficiency of ARK5.***A*, schematic diagram of full-length hnRNP A1, its fragment mutants, and F2 mutant. Fragments are not shown to scale; fragment 1 (1–86 aa), fragment 2 (87–211 aa), fragment 3 (212–320 aa), fragment 1 + 2 (1–211 aa), fragment 2 + 3 (87–320 aa), fragment 131 (131–320 aa), fragment 161 (161–320 aa). *B*, U2OS cells were transiently transfected with FLAG-hnRNP A1 WT (A1-WT) and fragments expressing plasmids, then FLAG-tagged proteins were immunoprecipitated and subjected to an *in vitro* kinase assay using PRECISIO GST-ARK5 kinase. *C*, U2OS cells were transiently transfected with WT or mutant FLAG-hnRNP A1 expressing plasmids, then FLAG-tagged proteins were immunoprecipitated, and subjected to an *in vitro* kinase assay using PRECISIO GST-ARK5 kinase. *D*, U2OS cells were transiently transfected with WT or mutant FLAG-hnRNP A1 expressing plasmids, then FLAG-tagged proteins were immunoprecipitated, and subjected to an *in vitro* kinase assay using mammalian expressed (U2OS) and in-house purified GST tagged ARK5 kinases (GST-ARK5-WT or GST-ARK5-KD). Recombinant GST-tagged SAMS peptide (SAMS) and GST were used as positive and negative controls, respectively. All the reactions were separated by SDS-PAGE and transferred to Immun-Blot PVDF membranes. The membranes were exposed to super sensitive phosphor screens and imaged in the Cyclone Plus storage phosphor system (Phosphor image above). Following imaging, the membranes were proceeded for immunoblotting and probed for respective antibodies (WB below). Proteins are indicated on the *right side* of the blots.

**Supplemental Figure S3:**
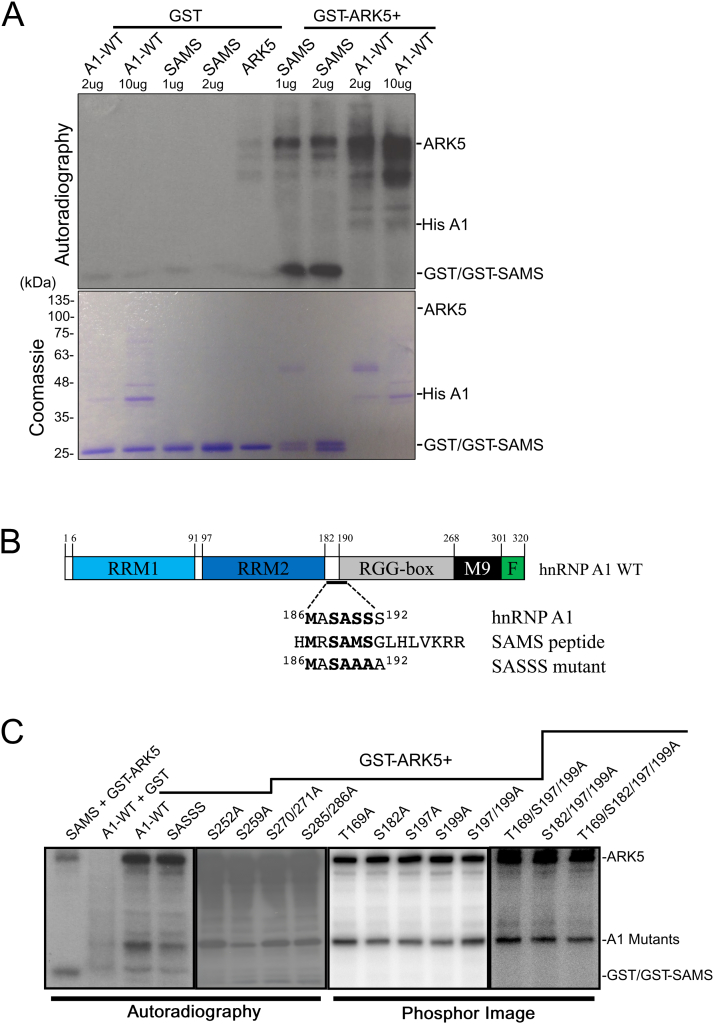
**ARK5 phosphorylates bacterial and mammalian expressed hnRNP A1.***A*, BL21 cells were transfected with pTrcHis plasmid expressing hnRNP A1 and the bacterially expressed hnRNP A1 protein (A1-WT) was isolated using His IMAC chromatography, subjected to an *in vitro* kinase assay using PRECISIO GST-ARK5, separated by SDS-PAGE, and exposed to autoradiography film (above). The parallelly run SDS-PAGE gel proceeded for Coomassie stain (shown below). Recombinant GST-tagged SAMS peptide (SAMS) was used as a positive control. *B*, schematic diagram of hnRNP A1 SASSS mutants used in the kinase assay. Not shown to scale. *C*, U2OS cells were transiently transfected with WT or indicated mutant FLAG-hnRNP A1 plasmids, then FLAG-tagged proteins were immunoprecipitated and subjected to an *in vitro* kinase assay using PRECISIO GST-ARK5, separated by SDS-PAGE, and exposed to autoradiography film or transferred in an ImmunBlot PVDF membrane, and exposed to Super Sensitive Phosphor Screens and imaged in the Cyclone Plus storage phosphor system. Proteins are indicated on the *right side* of the gels.

First, we examined whether ARK5 directly phosphorylates hnRNP A1. A radioactive *in vitro* kinase assay was performed using recombinant ARK5 and FLAG-tagged hnRNP A1 proteins purified from U2OS cells. GST-tagged SAMS peptide, a substrate shown previously to be directly phosphorylated by ARK5 (23), was used as a positive control, while GST served as a negative control. We observed that the full-length FLAG-tagged hnRNP A1 was robustly phosphorylated by ARK5 (Fig. 6*B*). However, one of the limitations of kinase assay using proteins purified from eukaryotic sources is the possibility of a coprecipitation of an unwanted, secondary protein attached to the protein of interest, which could provide a false positive signal in the assay (24). To confirm that the observed phosphorylation of hnRNP A1 is not due to the presence of an unknown protein that might have copurified with hnRNP A1, a bacterially expressed His-tagged hnRNP A1 was used instead of FLAG-tagged hnRNP A1. We observed distinct phosphorylation of bacterially expressed His-tagged hnRNP A1 by ARK5, although the signal appeared weaker than when using U2OS cell–expressed FLAG-hnRNP A1 (Fig. S3*A*). Thus, both data show that ARK5 directly phosphorylates hnRNP A1.

In order to identify hnRNP A1 residues that ARK5 phosphorylates, we generated deletion constructs of various lengths corresponding to different domains of hnRNP A1 (Fig. 6*A*), and these were subjected to *in vitro* kinase assay. Surprisingly, we observed that none of the individual fragments of hnRNP A1 (fragment 1 [1–86 aa], fragment 2 [87–211 aa], fragment 3 [212–320 aa]), could be phosphorylated by ARK5 (Fig. 6*B*). Similarly, the combined fragment 1 + 2 (1–211 aa) could not be phosphorylated, suggesting that the hnRNP A1 residues critical for the phosphorylation reside in the C-terminal portion of the protein. Indeed, we observed that fragment 2 + 3 (87–320 aa) was phosphorylated by ARK5 (Fig. 6*B*). We further truncated this fragment from the N terminus and produced two smaller fragments (fragment 131 [131–320 aa] and fragment 161 [161–320 aa]) (Fig. 6*B*). Both fragments were phosphorylated (Fig. 6*B*), suggesting that phosphorylation residues between 161 and 320 aa are the target for ARK5.

Since ARK5 phosphorylates fragment 161 (161–320 aa), but not fragment 3 (212–320 aa), we generated putative phosphor-null mutants by replacing all serines and threonines between 161 and 212 aa (T169, S182, S197, S199, and ^188^SASSS^192^) along with two randomly selected residues (S252 and S259) and S270/272 and S285/286 residues (selected from the M9 sequence) with alanine. Interestingly, SASSS region in the hnRNP A1 sequence has the sequence similarity with the SAMS peptide, which is a known target of ARK5 (Fig. S3*B*) (23). In addition, we also generated double, triple, and quadruple mutants (S197/199A, T169/S197/199A, S182/197/199A, T169/S182/197/199A) of putative phosphorylation sites in this region (Fig. S3*C*) and subjected them to an *in vitro* kinase assay. Notably, none of these mutants produced phosphorylation-null hnRNP A1 (Fig. S3*C*).

Having been unable to identify ARK5 phosphorylation residues of hnRNP A1, we turned our attention to the M9 and F-peptide regions that were previously shown to play a key role in the localization of hnRNP A1 (8, 11). To examine the role of these two domains of hnRNP A1 in phosphorylation by ARK5, we used the previously published ΔM9 and hnRNP A1 F2 mutant proteins (Fig. 6*A*) in the kinase assay (11, 15). Briefly, the hnRNP A1 F2 mutant has all serines of the F-peptide region mutated to alanine, whereas the ΔM9 variant is missing the M9 motif. In contrast to the WT hnRNP A1, we observed only weak phosphorylation signal on the ΔM9 and F2 mutants, similar to that of the GST negative control (Fig. 6*C*). This result shows that both the M9 motif and serines of F-peptide of hnRNP A1 can impact the phosphorylation efficiency of ARK5.

To confirm the specificity of ARK5 phosphorylation of hnRNP A1, we expressed and purified ARK5 WT and kinase-dead (KD) mutant (K84A) (25) from the mammalian cells (U2OS) and performed the kinase assay. We observed robust phosphorylation of hnRNP A1-WT and GST-SAMS (positive control) (Fig. 6*D*) and no phosphorylation signal on the ΔM9 (Fig. 6*D*) and F2 mutants (Fig. 6*D*) when using the ARK5-WT protein. In contrast, ARK5-KD was unable to phosphorylate any substrate (Fig. 6*D*). These data confirm the specificity of ARK5 as a kinase that phosphorylates hnRNP A1 directly, and also, M9 and F2 regions can impact the ARK5 phosphorylation efficiency.

**Figure 7:**
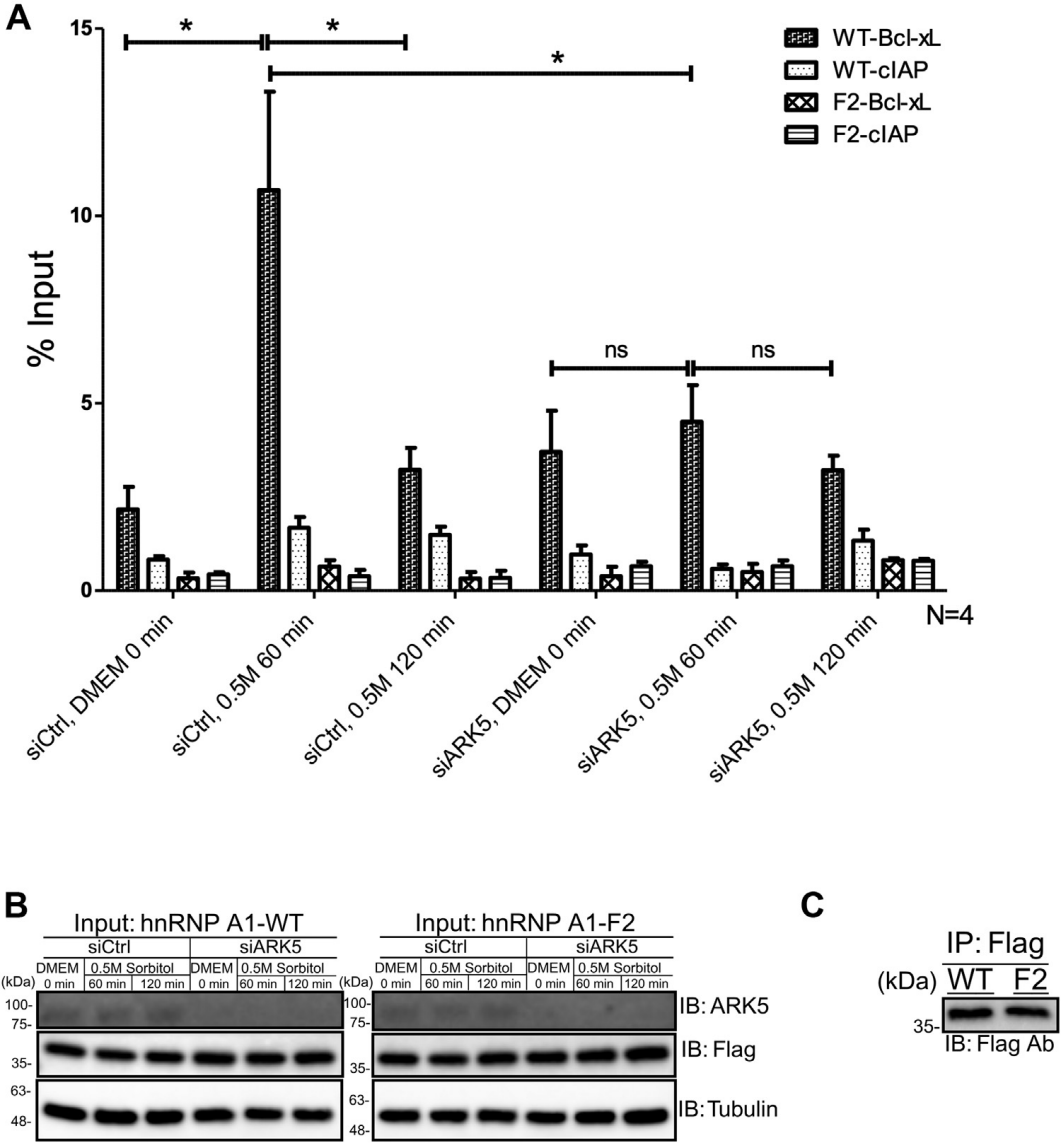
**Phosphorylated hnRNP A1 binds Bcl-xL mRNA during hypertonic stress.***A*, U2OS cells were transfected using siCTRL (negative control) or siARK5, followed by transfection of a Flag-tagged hnRNP A1 WT or F2 mutant plasmids. After 48 h, the cells were treated with either DMEM or 0.5 M sorbitol, harvested at the indicated time points and subjected to RNA-IP with Flag antibody. The associated RNAs were analyzed by reverse-transcriptase quantitative PCR (RT-qPCR) using Bcl-xL primer and cIAP primer (negative control). Normalized samples were presented as a percentage of the corresponding input. *B* and *C*, representative immunoblots of input samples (*B*) and immunoprecipitated samples (*C*) collected from (*A*). Tubulin was used as a loading control (∗*p* < 0.05; ns, not statistically significant). IP, immunoprecipitation.

We have shown that ARK5 and Bcl-xL have a reciprocal relationship, and it is also known that hnRNP A1 binds Bcl-xL mRNA (22). To understand how ARK5 and hnRNP A1 control Bcl-xL expression, we performed RNA immunoprecipitation (IP) experiments. U2OS cells were transfected with a negative siCTRL or siARK5 and then transfected with a Flag-tagged hnRNP A1-WT or hnRNP A1-F2 (a nonphosphorylatable mutant of hnRNP A1, Fig. 6*C*) plasmids, subsequently treated with DMEM or 0.5 M sorbitol, and samples were collected at different time points. The lysates were immunoprecipitated using Flag antibodies to capture Flag-hnRNP A1–RNA complexes; RNA was isolated and analyzed by reverse-transcriptase quantitative PCR using Bcl-xL and cIAP mRNAs (negative control, (17)) primers. We observed that hnRNP A1 interaction with Bcl-xL mRNA increased significantly during the first 60 min of sorbitol treatment and decreased after 120 min in the siCtrl samples. In contrast, there were no appreciable changes in the hnRNP A1-Bcl-xL mRNA binding in the siARK5-treated samples (Fig. 7*A*). Further, the hnRNP A1-F2 mutant did not bind Bcl-xL mRNA at all (Fig. 7*A*). These data suggest that phosphorylation of hnRNP A1 by ARK5 during hypertonic stress is needed for the binding of hnRNP A1 to Bcl-xL mRNA and that the F-peptide region of hnRNP A1 is critical for the mRNA binding.

### ARK5 directly interacts with hnRNP A1 in the nucleus

**Figure 8:**
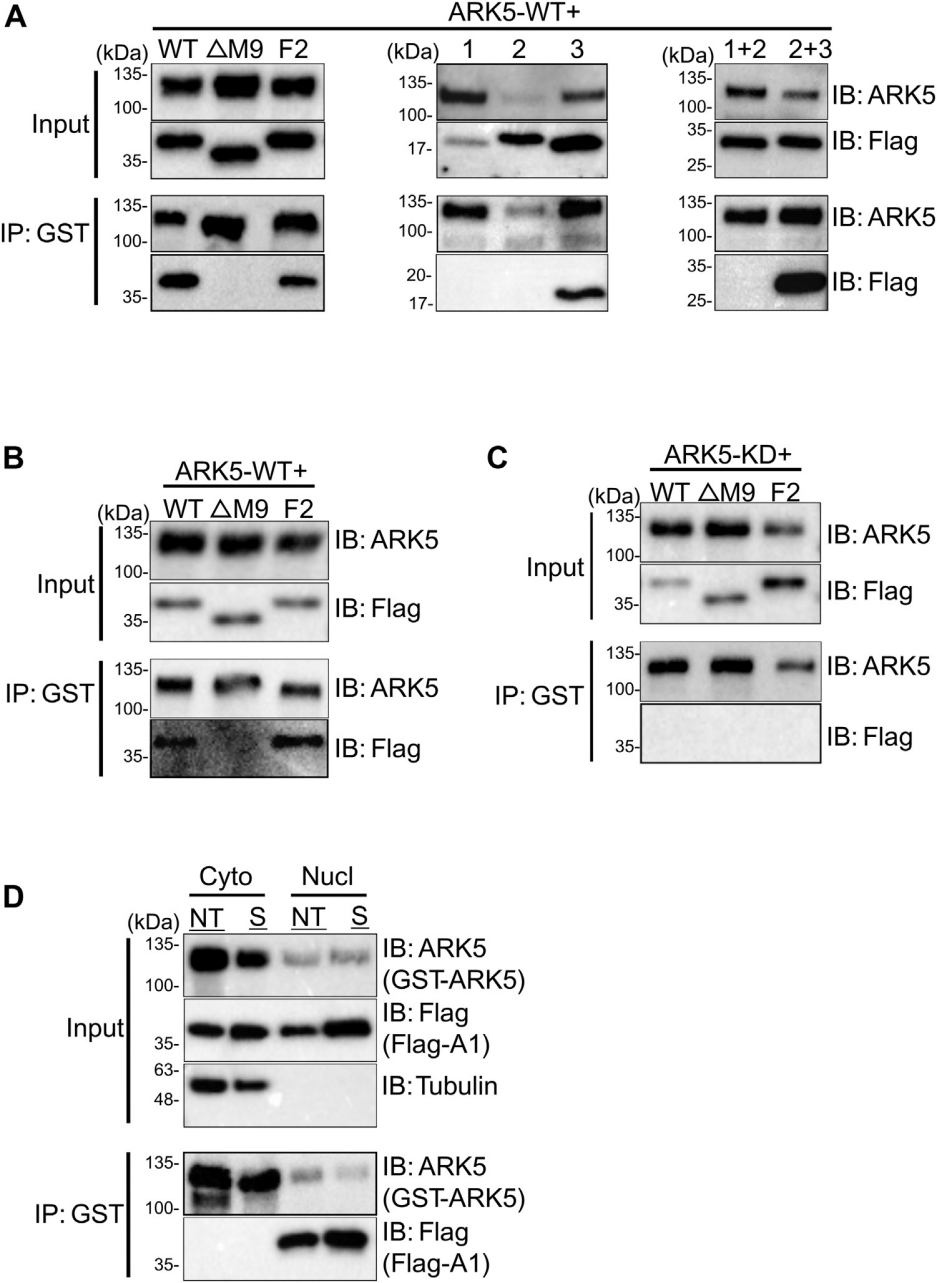
**ARK5 interacts with hnRNP A1 *via* M9 motif in the nucleus.***A*, *in vivo* coimmunoprecipitation of U2OS cells transfected with WT GST-tagged ARK5 (ARK5-WT) along with FLAG-tagged hnRNP A1 (WT, indicated fragments or mutants, see Table S1), and proceeded for GSH beads immunoprecipitation. *B*, *in vitro* coimmunoprecipitation of U2OS cells transfected with WT GST-tagged ARK5 (ARK5-WT) or with FLAG-tagged hnRNP A1 (WT) or mutants (△M9, F2), purified separately, and proceeded for GSH beads immunoprecipitation. *C*, *in vivo* coimmunoprecipitation of U2OS cells transfected with GST-tagged ARK5 kinase-dead (ARK5-KD) mutant along with FLAG-tagged hnRNP A1 (WT or mutants (△M9, F2)) and proceeded for GSH beads immunoprecipitation. Following gsh beads immunoprecipitation, the samples were separated by SDS-PAGE and proceeded for immunoblotting. *D*, subcellular fractionation of nuclear (Nucl) and cytoplasmic (Cyto) fractions of U2OS cells transfected with WT GST-tagged ARK5 (GST-ARK5) along with WT FLAG-tagged hnRNP A1 (FLAG-A1), and treated with DMEM (NT) or 0.5 M sorbitol (S). The fractions were proceeded for coimmunoprecipitation using glutathione beads and separated by SDS-PAGE and proceeded for immunoblotting. Proteins are indicated on the *right side* of the blots, and the respective molecular mass values are indicated on the *left side*. KD, kinase-dead.

**Supplemental Figure S4:**
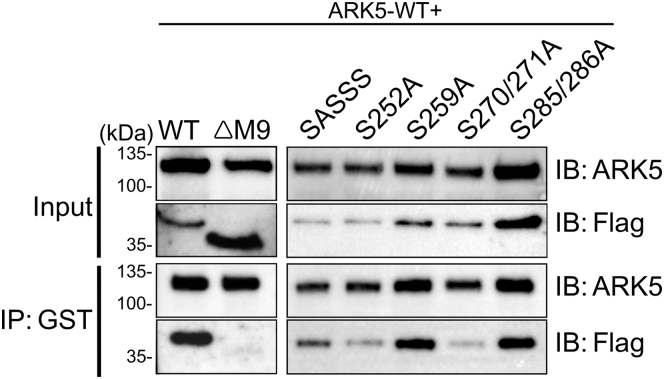
**ARK5 interacts with hnRNP A1 point mutants but not ΔM9.** Co-immunoprecipitation of U2OS cells transfected with indicated GST-tagged ARK5 (ARK5-WT) along with FLAG-tagged hnRNP A1 [wild-type (WT) or mutants] and proceeded for co-immunoprecipitation using glutathione beads and separated by SDS-PAGE and proceeded for immunoblotting. Proteins are indicated on the *right side* of the blots, and the respective molecular mass values are indicated on the *left side*.

We have shown that the M9 motif of hnRNP A1 is necessary for phosphorylation of hnRNP A1 by ARK5. However, the M9 motif is not known to be a target of phosphorylation; instead, this motif was previously shown to be involved in physical interaction between hnRNP A1 and transportins (12, 13, 14). It is therefore possible that the phosphorylation-null phenotype of the ΔM9 mutant is due to the lack of interaction with the kinase. Therefore, we wished to explore the nature of hnRNP A1-ARK5 interaction. *In vivo* co-IP was performed to identify the domains of interaction between these two proteins in FLAG-hnRNP A1 WT, ΔM9, and F2 mutants and GST-ARK5 cotransfected cells. We observed that WT and F2 mutant of hnRNP A1 interacted with ARK5, while ΔM9 mutant did not. (Fig. 8*A*). This result shows that the M9 motif is a necessary region for the interaction (Fig. 8*A*). We next used a series of hnRNP A1 fragments previously used in the kinase assays (Fig. 6*A*) to examine the role of M9 in interaction with ARK5. Indeed, only proteins harboring the M9 motif (full-length hnRNP A1, fragment 87–320, and fragment 212–320) were able to bind to ARK5 (Fig. 8*A*). In addition, hnRNP A1 mutants previously used in the kinase assay (Fig. S3*C*) were also examined for the ARK5 interaction using the similar *in vivo* co-IP experiment, and we found that all the 5 mutants interacted with ARK5 (Fig. S4). Collectively, these data confirm that M9 is the interaction domain between ARK5 and hnRNP A1.

Since ΔM9 and F2 mutants localize into different compartments of the cells (11), we performed an *in vitro* co-IP to determine whether the hnRNP A1 and ARK5 interact when purified separately. We observed that both WT and F2 mutant of hnRNP A1, but not ΔM9, interacted with ARK5 (Fig. 8*B*), providing evidence that stress-induced phosphorylation of hnRNP A1 is not required for the interaction. Next, we aimed to elucidate whether functional ARK5 is necessary for this interaction. We cotransfected FLAG-hnRNP A1 WT, ΔM9, or F2 plasmids with GST-ARK5-KD. We observed that none of the hnRNP A1 proteins interacted with the KD ARK5 mutant (ARK5-KD) (Fig. 8*C*). Taken together, our results demonstrate that functional ARK5 interacts directly with the hnRNP A1 through the M9 motif and that phosphorylation of the F-peptide is not required for this interaction.

**Supplemental Figure S5:**
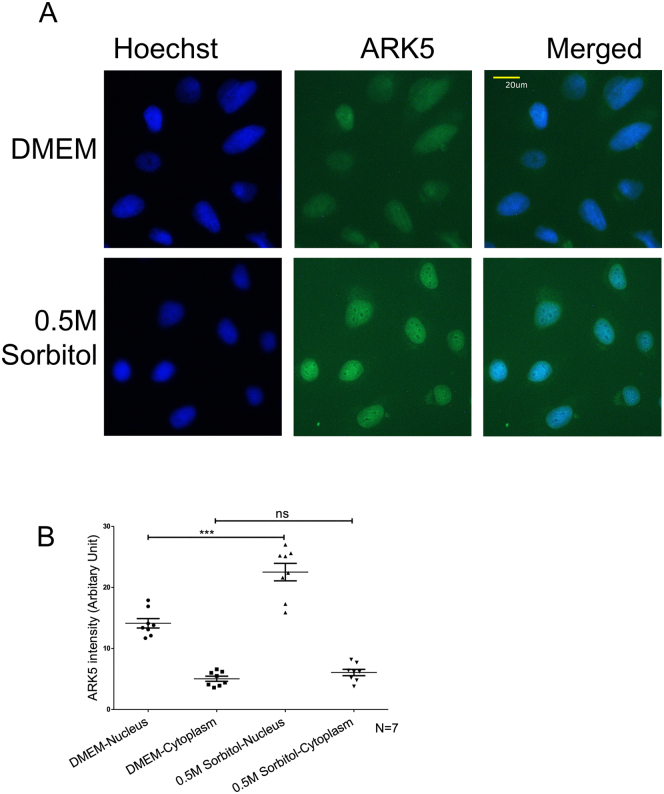
**ARK5 localizes in both the nucleus and cytoplasm, but mostly in the nucleus during hypotonic stress.***A*, representative immunofluorescence microscopy images of U2OS cells treated with DMEM (*top row*) or a 0.5 M sorbitol for 2 h (*bottom row*). Cells were stained with ARK5 antibody. Nuclei were visualized using Hoechst staining. Images were taken using the 40× objective. Scale bar shown in the *top right image* pertains to all images. *B*, quantification of nuclear and cytoplasmic ARK5 intensity for DMEM *versus* hypertonic shock treated cells of replicates of *A* using Fiji image analysis software (∗∗∗*p* < 0.001).

The observation that the M9 domain of hnRNP A1 (the domain responsible for cytoplasmic retention of hnRNP A1 (26)) is required for the binding to ARK5 prompted us to investigate in which cellular compartment the interaction between ARK5 and hnRNP A1 takes place. To this end, cytoplasmic and nuclear fractions of U2OS cells cotransfected with FLAG-hnRNP A1 and GST-ARK5 were subjected to co-IP using GSH beads. We observed that hnRNP A1 interacts with the ARK5 exclusively in the nucleus (Fig. 8*D*). To understand the distribution of ARK5 in U2OS cells during hypertonic stress, we performed immunofluorescence staining and imaging, which shows that ARK5 is distributed in both nucleus and cytoplasm but with relatively higher concentration in the nucleus (Fig. S5).

## Discussion

Cellular stress causes changes in signaling pathways to facilitate adaptation to the new environment or to induce programmed cell death (1). The cytoplasmic accumulation of hnRNP A1 is one of the consequences of cellular stress response and is regulated by its modifications, primarily by phosphorylation of key residues within the C terminus of the protein. We used RNAi-based kinome-wide screen, which is a genetic approach to identify the kinase(s) of hnRNP A1 in the complex signaling pathways involved in the hypertonic stress. We have identified several candidate kinases that regulate the cytoplasmic accumulation of hnRNP A1. Through the kinome-wide screen, we had previously characterized the HK2 as a kinase of hnRNP A1 and also confirmed the involvement of p38β (MAPK11), but not the other three p38 MAPK isoforms (p38α (MAPK14), p38γ (MAPK12) and p38δ (MAPK13)), for cytoplasmic accumulation of hnRNP A1 (15). Thus, HK2, MAPK11 (p38β), Mnk1/2, and MKK_3/6_-p38 kinases are already identified as regulators of hnRNP A1’s subcellular localization (11, 15, 16, 17, 19, 27). However, none of these kinases were shown to be interacting with hnRNP A1 directly, and for several of them (*e.g.*, HK2, MAPK11, MKK_3/6_-p38), the direct phosphorylation of hnRNP A1 by these kinases was also not demonstrated. Here, we demonstrate that ARK5 as another regulator for cytoplasmic accumulation of hnRNP A1 in response to hypertonic stress.

hnRNP A1 plays many roles in protein synthesis and RNA metabolism. One role of relevance to this study is the regulation of the expression of select proteins, particularly those related to cell viability. It has been shown that hypertonic stress-induced cytoplasmic accumulation of hnRNP A1 regulates the IRES-mediated expression of Bcl-xL (19). Specifically, following the exposure to hypertonic stress and concomitant cytoplasmic accumulation of hnRNP A1, Bcl-xL levels increase during early time points but will dissipate over the next several hours as IRES-translation is repressed (19). Our results suggest that ARK5 is a negative regulator of Bcl-xL, and also, during the hypertonic stress, ARK5 promotes the activation of caspases 3/7 in comparison to the siARK5-treated cells. In contrast to these results, it has been noted in the literature that during periods of stress brought on by nutrient starvation, ARK5 directly phosphorylates caspase 6 and inhibits its activation (28, 29), suggesting that different cellular stresses trigger distinct response pathways. In contrast, our data suggest a model where ARK5 regulates Bcl-xL expression through hnRNP A1 and consequently attenuates the mitochondrial caspase cascade. These two different mechanisms likely contribute to increased plasticity of the cellular stress response. Indeed, ARK5 plays an important role in cell survival, tumor progression, invasion, and metastasis (30, 31). For example, ARK5’s expression is deregulated in several cancers, including colon, pancreas, breast, and various gliomas (31, 32, 33). Some of these roles have been noted to be triggered by stressors, such as nutrient deprivation, which induces the activation of ARK5 through phosphorylation by Akt at Ser560 and LKB1 at Thr211, leading to ARK5 interacting with targets such as PP1β^MYPT1^, p53, and caspase 6 (25, 28, 30).

Allemand *et al.* (11) has shown that, during hypertonic stress, hnRNP A1 is phosphorylated on the serine residues of the C-terminal F-peptide, which forces the protein to accumulate in the cytoplasm, but the kinase had not been identified. It has also been shown that Mnk1/2 kinases phosphorylated full-length hnRNP A1 (although the precise phosphorylation region was not identified) and that reduction of Mnk1/2 levels by RNAi or expression of dominant-negative mutant proteins abolished hnRNP A1 cytoplasmic accumulation upon hypertonic stress (11, 15, 16, 17, 19, 27). Another study has shown that in response to T-cell activation Mnk1 kinase phosphorylated hnRNP A1 on Ser192 and Ser310/311/312 of F-peptide (34). Unlike hypertonic stress, T-cell activation has shown no evidence of cytoplasmic accumulation of hnRNP A1 presumably because the phosphorylation sites on hnRNP A1 in response to these distinct stimuli are different (34). Therefore, while the available literature does not support Mnk kinases as primary kinases that phosphorylate F-peptide in response to hypertonic stress, our data show the strong link between ARK5 kinase and the phosphorylation of hnRNP A1 at the F-peptide region during hypertonic stress. We have previously attempted to detect phosphorylation sites of recombinant hnRNP A1 using mass spectrometry but were unable to detect the F-peptide fragment. This is likely due to the fact that multiphosphorylated peptides are relatively hydrophilic and do not adhere to standard C18 trapping columns prior to mass spectrometry analysis, making them difficult to detect (35).

Interestingly, our data suggest that M9 is the interaction domain between ARK5 and hnRNP A1. The C-terminal region of hnRNP A1, which includes a glycine-rich domain, and the M9 motif has a low-complexity domain structure similar to prion domains (PrDs), and the C-terminal region is also known as the prion-like domain (PrLD) of hnRNP A1 (36). The PrLDs in human RBPs including hnRNP A1 play important roles in the functional protein–protein interactions (36). In addition to the role of M9 motif in the hnRNP A1 and transportin-1/2 interaction (14), Duan *et al.* (37) has shown that poly(ADP-ribosyl)ation (PARylation) of hnRNP A1 at K298 (one of the residues of the M9 motif) controls its nucleocytoplasmic transport and also promotes the hnRNP A1 and TDP-43 interaction. In addition, our data also suggest that the hnRNP A1 interacts with the ARK5 in the nucleus. The nuclear interaction of ARK5 kinase with its target is also supported by another research, in which ARK5 was shown to bind with and phosphorylate p53 (Ser15/392) in the nucleus upon glucose starvation (25). Indeed, the human protein atlas also indicates that ARK5 expression and localization is cell line specific, in which U2OS and U-251MG cells show the localization in nucleoplasm, nucleoli fibrillar center, and microtubules, whereas A431 cells show the localization only in nucleoplasm and nucleoli fibrillar center (38).

Our work adds ARK5 to the list of kinases which phosphorylate hnRNP A1 and therefore regulates its localization in response to stress. Of note, all four kinases (HK2, MAPK11, Mnk1/2, and ARK5) seem to play the same role during hypertonic stress. It is therefore possible that these kinases may be arrange hierarchically or could respond to distinct cues that are not easily discernible in the laboratory-induced hypertonic stress. Additionally, both ARK5 and MAPK11 kinases are activated by hypertonic shock, suggesting an operational link between AMPK and MAPK signaling pathways. However, of these kinases, ARK5 is the only kinase identified as directly interacting with hnRNP A1. Also, our data show that ARK5 interacts with hnRNP A1 in the nucleus, suggesting that hnRNP A1 is phosphorylated by ARK5 in the nucleus. The phosphorylation of hnRNP A1 enhances its binding to target mRNAs, such as Bcl-xL, which could subsequently impact their nuclear export, similarly to what was proposed for FGF-2/S6K2 signaling axis (17). Overall, our work adds to the discovery of novel regulatory processes critical for cellular stress response and survival by determining the impact of ARK5 on hnRNP A1-mediated posttranscriptional events and consequent modulation of the cellular stress response.

## Experimental procedures

### Reagents, siRNA, primers, and antibodies

See Supplementary Materials.

### Cell culture and transfection

Human osteosarcoma U2OS cells were cultured in complete Gibco high glucose DMEM (Thermo Fisher Scientific) supplemented with 10% heat-inactivated fetal bovine serum, 1% L-glutamine, 1 × 10^5^ U/l penicillin, and 100 g/l streptomycin at 37 °C with 5% CO_2_. For the siRNA-mediated knockdowns, 2 × 10^5^ U2OS cells were seeded into the wells of a 6-well culture plate and cultured in complete DMEM for a period of 24 h. Following this, cells were transfected using Lipofectamine RNAiMAX transfection reagent (Invitrogen) and the manufacturer’s protocol with 20 nM of siRNAs and maintained for 24 h in DMEM without penicillin/streptomycin (P/S) and then proceeded for further experiments as explained below. For the overexpression of specific proteins, cells were seeded and cultured in an identical manner as knockdowns during the first 24 h. Subsequently, cells were transfected with 1 μg of desired plasmids as per experiment using Lipofectamine 3000 transfection reagent (Invitrogen) and the manufacturer’s protocol. Transfected cells were maintained in DMEM without P/S for 4 h, at which point the media containing the plasmid in question were removed and fresh DMEM without P/S was added, without rinsing the wells, then subsequently incubated for additional 20 h, and then processed for further experiments as outlined later. For rescue experiments, cells were seeded at 1 × 10^5^ cells per well onto a 6-well culture plate, then maintained for 24 h. Cells were then transfected using Lipofectamine RNAiMAX transfection reagent (Invitrogen) and the manufacturer’s protocol with 20 nM of siRNAs and maintained for 24 h in DMEM without P/S followed by the subsequent overexpression, in which cells were transfected with 1 μg of desired plasmids as per experiment using Lipofectamine 3000 transfection reagent and the manufacturer’s protocol. Transfected cells were maintained in DMEM without P/S for 4 h, at which point the media containing the plasmid in question was removed and fresh DMEM without P/S was added, without rinsing the wells, then subsequently incubated for additional 20 h, and then processed for further experiments as outlined later.

### Hypertonic stress

Hypertonic stress was induced by sorbitol as described previously (15). Briefly, cells were rinsed with 1× PBS, followed by the subsequent addition of either warmed complete DMEM media or 0.5 M D-Sorbitol (Sigma–Aldrich) solution in complete DMEM. The 0.5 M D-Sorbitol (500 mOsm/l), in addition to DMEM (300 mOsm/l), has a total osmolarity of 800 mOsm/l, which is considered extreme osmotic stress (39). However, the response to sorbitol also depends on the cell types (40). The acute stress was performed at 37 °C with 5% CO_2_ for 2 h, at which point media was removed and cells were rinsed with 1× PBS, then collected as described later.

### Plasmids, cloning, and mutagenesis

See Table S1 for a detailed list of plasmids and primers.

FLAG-tagged hnRNP A1 fragments were created using a FLAG-hnRNP A1 WT as the template, and a PCR was performed using PfuUltra II Fusion HS DNA polymerase (Agilent) using the primers described in the Table S1. The PCR products were digested using the EcoRI and XbaI (Invitrogen) to create the desired insert. For hnRNP A1 point mutants and the GST-tagged ARK5-KD mutant, site-directed mutagenesis was performed by PCR using PfuUltra II Fusion HS DNA polymerase or the Q5 Site-Directed Mutagenesis Kit (NEB) and the templates and primers as described in the Table S1. All constructs were confirmed by sequencing at StemCore Laboratories, OHRI.

### Protein purifications

GST-tagged proteins expressed in bacteria: BL21(DE3) *Escherichia coli* expressing the GST-tagged SAMS peptide or GST were grown for 24 h and pelleted by centrifugation at 5000*g* for 15 min at 4 °C. Cell pellets were suspended in 10 ml of cold PBS and centrifuged again, after which the cell pellets were suspended in 10 ml of lysis buffer (50 mM Tris–HCl pH 8.0, 200 mM NaCl, 1 mM EDTA, ddH_2_O supplemented with 1 mM DTT, and 2 mM PMSF). Lysates were then sonicated on ice at 12% amplification for two sets of 10 s with 1 min break. Triton X-100 (100 μl) was added to each lysate, then centrifuged at 2200*g* or 10 min at 4 °C and supernatants were collected. Two hundred microliters of a 50% slurry of GSH Sepharose 4 Fast Flow beads (Sigma–Aldrich) was added, then lysates were set to rotate for 2 h at 4 °C. Once collected, lysates were centrifuged at 500*g* for 5 min and then rinsed five times with 5 ml of PBS. Finally, beads were suspended in 1 ml of PBS and the proteins were eluted with the addition of 600 μl of L-glutathione pH 8.0, while rotating at 4 °C for 1 h. Protein concentration was quantified using Bradford assay. Samples were aliquoted, equal volume 20% glycerol mixed into 2× TKGD buffer (20 mM Tris–HCl pH 7.4, 200 mM KCl, and 1% glycerol, supplemented with 1 mM DTT) was added, and stored at −80 °C.

#### His-tagged protein expressed in bacteria

BL21(DE3) *E. coli* expressing the His-tagged hnRNP A1 was grown for 24 h and pelleted by centrifugation at 3500*g* or 15 min at 4 °C, then lysed in 20 ml of TKGD buffer supplemented with 1 mM DTT, two tablets of cOmplete protease inhibitor cocktail (Sigma–Aldrich), and 2 mg/ml of lysozyme. Samples were kept on ice for 30 min prior to adding 5 μl DNAse 1 enzyme and 300 μl of 1 M MgCl_2_. Samples were sonicated at 25% amplification, six times for an interval of 30 s on, 30 s off. Triton X-100 (0.5 ml) was added to each sample, then set to rotate at 4 °C for 30 min. Samples were centrifuged at 2200*g* for 30 min at 15 °C and supernatant was collected. About 1 M imidazole (0.25 ml) solution prepared in TKGD buffer was added to each sample, then loaded, one sample at a time, into a HisTrap HP column (Sigma–Aldrich) following the manufacturer’s protocol. His-tagged proteins were step eluted by increasing concentrations of imidazole solution and each elute was collected, tested *via* SDS-PAGE, then Coomassie stained to identify the eluate with the highest purity of the protein, which was subsequently dialyzed overnight in TKGD buffer at 4 °C, then passed through an Amicon Ultra centrifugal filter tube (Sigma–Aldrich) to concentrate. Samples were aliquoted, equal volume 20% glycerol mixed into 2× TKGD buffer was added, and stored at −80 °C.

#### FLAG-tagged proteins expressed in mammalian cells

U2OS cells were transiently transfected with the desired plasmids expressing FLAG-tagged proteins for 24 h as described previously; cells were rinsed with PBS, then scraped from the wells, recovered in 1 ml of PBS, and transferred to an Eppendorf 1.5 ml tube. Cells were centrifuged at 16,000*g* for 1 min at room temperature (RT), then supernatant was discarded. Cell pellets were suspended in 500 μl of lysis buffer (50 mM Tris–HCl pH 7.4, 150 mM NaCl, 1 mM EDTA, 1% Triton X-100, and supplemented with cOmplete Protease inhibitor cocktail (Sigma–Aldrich)), then sonicated at 20% amplification for 6 s and centrifuged for 20 min at 16,000*g* at 4 °C. Following the spin down, 50 μl of each sample were set aside at −20 °C for immunoblotting. Prepared Anti-FLAG M2 Affinity Gel (Sigma–Aldrich) beads (30 μl) were added to each sample and then set to rotate for 2 h at 4 °C. Samples were then centrifuged at 3500*g* for 2 min at 4 °C, subsequently supernatant was discarded and the beads rinsed in 1 ml of Tris-buffered saline (TBS). This last step was repeated three times. Beads were then rinsed with 1× kinase buffer (200 mM Tris–HCl pH7.5, 50 mM β-glycerophosphate, ddH_2_O, supplemented with 2 mM Na_3_VO_4_ and 2.5 μM DTT), then the supernatant was discarded, and the affinity gel was used in *in vitro* kinase assays.

#### GST-tagged proteins expressed in mammalian cells

U2OS cells were transiently transfected with desired plasmids expressing GST-tagged proteins for 24 h as described previously; cells were rinsed with PBS, then scraped from the wells, recovered in 1 ml of PBS, and transferred to an Eppendorf 1.5 ml tube. Cells were centrifuged at 16,000*g* for 1 min at RT, then supernatant was discarded. Cell pellets were suspended in 500 μl of lysis buffer (50 mM Tris–HCl pH 7.4, 150 mM NaCl, 1 mM EDTA, 1% Triton X-100, and supplemented with cOmplete protease inhibitor cocktail (Sigma–Aldrich)), then sonicated at 20% amplification for 6 s and centrifuged for 20 min at 16,000*g* at 4 °C. Following the spin down, the supernatants were purified in the small scale using Pierce GST spin purification kit and manufacturer’s protocol. Finally, eluted proteins were concentrated using 10K MWCO Pierce protein concentrator, and the protein concentration was quantified using Bradford assay. Proteins were stored in kit’s elution buffer with 25% glycerol, aliquoted, and stored at −80 °C.

### Protein extraction and Western blotting

For protein extraction, cells were rinsed twice with PBS, then scraped from their wells using a cell lifter, transferred to an Eppendorf tube in PBS, and centrifuged at 16,000*g* for 1 min at RT. Supernatant was discarded, the cell pellets suspended in 50 or 100 μl of radioimmunoprecipitation assay lysis buffer (50 mM Tris–HCl pH 7.4, 1 mM EDTA, 150 mM NaCl, 1% NP-40, 0.5% SDS, and 0.5% deoxycholic acid supplemented with either cOmplete protease inhibitor cocktail (Sigma–Aldrich) or 1 μM PMSF) and samples placed on ice for 30 min followed by 15 min centrifugation at 18,500*g* at 4 °C. Supernatant was collected and the concentration of proteins was calculated using a Bradford assay. Protein lysates (30 μg) were mixed with ddH_2_O and prepared 2× Laemmli sample buffer (Bio-Rad) (supplemented with 5% β-mercaptoethanol), then set to migrate on a 10% SDS-PAGE gel. Electrophoresis was performed at 180 V for 50 min, then wet transfer was performed for 80 min at 100 V onto 0.2 μm Immun-Blot polyvinylidene difluoride (PVDF) membrane (Bio-Rad Laboratories Inc). Membranes were then probed for proteins using antibody conditions found in Table S1. Following the antibody incubation periods, membranes were rinsed with TBS or PBS, based on what was used during the antibody incubations, then incubated in Pierce ECL Western Blotting substrate (Thermo Fisher Scientific) to render the probed proteins visible once exposed onto HyBlot CL Autoradiography Film (Denville) when developed using a Kodak X-OMAT 2000A processor or exposed the blot using ChemiDoc MP (Bio-Rad).

### *In vitro* kinase assay

Kinase assays were performed using proteins purified as described previously. Per sample, 2 μg of substrate (or 10 μl of affinity gel), 3 μl of kinase buffer (200 mM Tris–HCl pH7.5, 50 mM β-glycerophosphate, ddH_2_O, supplemented with 2 mM Na_3_VO_4_ and 2.5 μM DTT), 0.2 μg of baculovirus expressed kinase (NUAK1, GST-tagged, Human, PRECISIO Kinase, Recombinant, Sigma–Aldrich, catalog no.: #SRP5237) or 0.2 μg of mammalian cells expressed kinase or 2 μg of negative control protein (GST), and up to 20 μl of ddH_2_O were combined. Samples were then mixed with 10 μl of ^32^P-ATP master mix (1 μl Gamma ^32^P-ATP (EasyTide, PerkinElmer Ltd), 3 μl 10× M-ATP (300 μM ATP, 66 mM MgCl_2_, 33 mM MnCl_2_, ddH_2_O), and 6 μl ddH_2_O). This mixture was then vortexed, centrifuged at 16,000*g* for 10 s, and incubated for 30 min at 30 °C. 4× SDS loading dye (10 μl) was added to the mix, gently vortexed, centrifuged, then boiled at 95 °C for 5 min, after which the samples were centrifuged again. Samples were pipetted into the wells of a 10% SDS-PAGE gel, then electrophoresis proceeded for 40 min at 180 V. Gels with samples were either dyed with Coomassie stain or wet transferred onto a PVDF membrane as described previously. The stained gels or membranes were then sealed in cellophane, mounted onto a film cassette, along with a BioMax Transcreen High Energy Intensifying Screen (Kodak) and Amersham Hyperfilm ECL High Performance Chemiluminescence Film (GE Healthcare), then stored at −80 °C for the desired time required to obtain visualization of bands (no longer than 24 h). Alternatively, the PVDF membranes were sealed in cellophane, mounted onto a film cassette, along with a BioMax Transcreen High Energy Intensifying Screen (Kodak) and Cyclone Super Sensitive Phosphor Screen (PerkinElmer), then exposed for 2 h and subsequently scanned and imaged in the Cyclone Plus storage phosphor system using OptiQuant software (PerkinElmer) to obtain visualization of bands.

### Immunofluorescence and confocal microscopy

Cells were seeded onto sterilized coverslips (22 × 22 mm, #1 thickness, VWR) mounted in the wells of a 6-well culture plate. Once the desired experimental treatment was completed, the wells were rinsed once in PBS, then the coverslips were collected and set aside into a sterile 6-well culture plate for further treatment, while the remainder of the cells were collected for immunoblot analysis as described previously. Once removed, coverslips were fixed using a 3.7% formaldehyde solution (diluted in PBS) for 15 min with gentle rocking, then rinsed with PBS. Subsequently, coverslips were incubated in a 0.2% Triton X-100 solution (diluted in PBS) for 5 min with gentle rocking, then rinsed three times with PBS. Cells were then incubated in a 1% fetal bovine serum solution (diluted in a Triton X-100/bovine serum albumin buffer solution [0.2% bovine serum albumin, 0.004% Triton X-100, PBS]) for 15 min with gentle rocking, serving as the blocking agent, then stored at 4 °C overnight. The following day, wells were incubated using the antibody conditions described in Table S1. Wells were rinsed three times in PBS between antibody treatments, and while the secondary antibody was applied, the culture plate was wrapped in tinfoil to shield from exposure to light. Coverslips were finally treated with 1 μg/ml of Hoechst 33342 solution (diluted in PBS) (Invitrogen) for 5 min with gentle rocking. Coverslips were then rinsed three times for 5 min with PBS and subsequently mounted onto Superfrost Microscope Slides (25 ×75 × 1.0 mm, Thermo Fisher Scientific Co) using Dako fluorescent mounting medium (Agilent), then set aside in a dark area to dry before applying a sealant over the edges of the coverslips. Images were taken using the Olympus 1X81 confocal microscope while using the 60×, water-based objective.

### Kinetic cell imaging

Following the rescue experiment treatment, cells on the 6-well culture plates were rinsed once with PBS, then either 1.5 ml of complete DMEM or 0.5 M D-Sorbitol solution was added to the desired wells. Both complete DMEM and 0.5 M D-Sorbitol solution were supplemented with the IncuCyte Caspase 3/7 reagent for apoptosis (Essen BioScience), at a dilution of 1:5000 from the stock. Using the IncuCyte Zoom Live Kinetic Imaging System (Essen BioScience) and related software, green immunofluorescence was measured each hour over a period of 10 h. Raw data were then exported and analyzed *via* Microsoft Excel and GraphPad Prism 6 software (GraphPad Software Inc).

### ARK5 drug-based inhibition

Following the culturing and seeding of 2 × 10^5^ U2OS cells onto a 6-well culture plate mounted with coverslips (22 × 22 mm, #1 thickness, VWR) for 24 h as described previously, cells were rinsed with PBS. As pretreatment, each well was treated with either 2 μl of dimethyl sulfoxide (DMSO) or 2 μl of WZ4003 (Tocris, Bio-Techne) (diluted in DMSO), final concentration of 10 μM, diluted in 2 ml of complete DMEM for 4 h at 37 °C with 5% CO_2_. After which, cells were rinsed with PBS, then treated again with an identical amount of DMSO or drug diluted in either complete DMEM or 0.5 M D-Sorbitol solution for 2 h. Following the stress period, coverslips were collected and treated as described in the immunofluorescence section and the remainder of the cells in the wells was collected using the manner described in the protein extraction protocol.

### Co-IP

U2OS cells were transiently transfected with the desired plasmids for 24 h as described previously; the cells were rinsed with PBS, then scraped from the wells, recovered in 1 ml of PBS, and transferred to an Eppendorf 1.5 ml tube. Cells were centrifuged at 16,000*g* for 1 min at RT, then supernatant was discarded. Cell pellets were suspended in 500 μl of co-IP buffer (25 mM Tris–HCl pH 7.4, 150 mM NaCl, 50 mM NaF, 0.5 mM EDTA, 0.5% Triton X-100, 5 mM β-glycerophosphate, 5% glycerol, and ddH_2_O), supplemented with cOmplete Protease Inhibitor Cocktail (Sigma–Aldrich Co), and then set to rotate for 2 h at 4 °C. Cells were then centrifuged for 20 min at 16,000*g* at 4 °C. Following the spin down, 50 μl of each sample were set aside at −20 °C for immunoblotting. Prepared GSH Sepharose 4 Fast Flow beads (Sigma–Aldrich) (50 μl) were added to each sample, then set to rotate for overnight at 4 °C. Samples were then centrifuged at 3500*g* for 2 min at 4 °C, subsequently supernatant was discarded and the beads rinsed in 1 ml of co-IP buffer (minus protease inhibitor cocktail supplementation). The last step was repeated five times. Rinsed beads were suspended in 20 μl of TBS before adding to an SDS-PAGE gel for immunoblotting as described previously.

### Subcellular fractionation

Nuclear and cytoplasmic fractions were isolated using a CNM compartmental protein extraction kit according to the manufacturer’s protocol (BioChain Inc). U2OS cells were transiently transfected with the desired plasmids for 24 h as described previously; the cells were rinsed with ice-cold PBS twice, then scraped from the wells, recovered in 1 ml of PBS, and transferred to an Eppendorf 1.5 ml tube and pelleted. Cell pellets were suspended in 150 μl of ice-cold buffer C, and the mixture was rotated at 4 °C for 20 min. The cell mixture was passed through the needle base of a 26.5 gauge needle ∼50 times to disrupt the cell membrane and release the nuclei from the cells. After centrifugation at 15,000*g* and 4 °C for 20 min, the cytoplasmic proteins in the supernatant were collected. The pellet was suspended in 150 μl of ice-cold buffer W, and the mixture was rotated at 4 °C for 5 min. The supernatant was centrifuged at 15,000*g* and 4 °C for 20 min, and the pellet was suspended in 75 μl of ice-cold buffer N followed by rotation at 4 °C for 20 min. After centrifugation at 15,000*g* at 4 °C for 20 min, the nuclear proteins in the supernatant were collected. The collected nuclear and cytoplasmic fractions were co-IP using GSH Sepharose 4 Fast Flow beads as described previously and analyzed by SDS-PAGE electrophoresis and immunoblotting as described previously.

### RNA-IP

RNA-IP was conducted as described previously (41). U2OS cells were transfected using the desired siRNA for 24 h and then transfected with the indicated plasmids for 24 h, as described previously. After 48 h, the cells were treated with either DMEM or 0.5 M sorbitol and then harvested at different time points; then, Flag-hnRNP A1 was immunoprecipitated from the cell lysates using Anti-FLAG M2 Affinity Gel. Total RNAs were isolated from the pull-down samples using RNAzol (Sigma–Aldrich), and the associated mRNAs were transcribed to complementary DNAs using the iScript complementary DNA synthesis kit (Bio-Rad). Quantitative PCR was performed using gene-specific primers, SsoAdvanced Universal SYBR Green Supermix (Bio-Rad), and CFX96 Real-Time System (Bio-Rad). The primers information is in Table S1. The quantification cycle (Cq) of mRNAs was analyzed with CFX Maestro1.1 software (Bio-Rad). Each sample was normalized to its respective input and presented as a percentage input, as described previously (42).

### Statistical analysis

Data collection for immunofluorescent images was performed using Columbus image analysis program (PerkinElmer) as described in (15) or Fiji image analysis software (43). Caspase-3/7 analysis and data collection was performed using Essen BioScience IncuCyte Zoom kinetic cell imager and the related software. Densitometry of immunoblots was analyzed using LI-COR Image Studio software and BioRad Image Lab software. Where appropriate, data were analyzed using one-tailed unpaired Student *t* test with Welch’s correction using the GraphPad Prism 6 software and are expressed as a mean ± SEM of at least three independent experiments.

## Data availability

All data are contained in the article and is available upon request.

## Supporting information

This article contains supporting information (15).

## Conflict of interest

The authors declare that they have no conflicts of interest with the contents of this article.
