# Supplementary material for: AMPK-related protein kinase ARK5 regulates subcellular localization of RNA-binding protein hnRNP A1 during hypertonic stress
Source: J Biol Chem. 2022 Aug 11;298(9):102364. doi: 10.1016/j.jbc.2022.102364 (PMC9478406; doi:10.1016/j.jbc.2022.102364)
Supplement: Supplemental Table S1 [file mmc1.docx]

**Supplementary Table 1**

| **Plasmids Information** | | |
| --- | --- | --- |
| **Construct** | **Vector** | **References** |
| Flag-hnRNPA1 Wild-Type | pCI | Lewis et al., 2007 |
| His-hnRNP A1 Wild-Type | pTrcHis | N/A |
| GST-ARK5 Wild-Type | pDEST27 | Provided by Dr. Screaton, Sunnybrook Health Sciences Centre |
| GST-ARK5 Kinase-Dead | pDEST27 | Humbert et al., 2010 |
| Flag-hnRNP A1 F2 | pCI | Allemand et al., 2005(Provided by Dr. Stephen Lewis, ACRI Moncton, NB) |
| Flag-hnRNP A1 ΔM9 | pCI | Allemand et al., 2005(Provided by Dr. Maria Hatzoglou, Clevland, OH) |
| GST and GST-SAMS | pGEX4T-1 | Provided by Dr. Tsuchihara, NCC EPOC |
| Flag control | pcDNA3 | Lewis et al., 2007 |
| Kate EV | pmKate2-C | N/A |

| **siRNA Target Sequences (5’-3’) and RT-qPCR primers:** | | | |
| --- | --- | --- | --- |
| **Gene** | **Primer name** | **Target Sequence (5’-3’)** | **Cat. # (Company)** |
| Control | Negative Control | AAUUCUCCGAACGUGUCACGU | 1027310 (Qiagen) |
| NUAK1 | siARK5#1 | AGGAGUGCUGUGAUUGACUAGUAAU | Supplied by Dr. Lewis (ACRI) |
| NUAK1 | siARK5#2 | CGGCAGGACUCUUAUCUUA | J-004931-12 (Dharmacon) |
| BCL2L1 | Bcl-xL | N/A | QT00236712 (Qiagen) |
| cIAP | cIAP | GCCTGATGCTGGATAACTGG(Forward) GGCGACAGAAAAGTCAATGG(Reverse) | IDT (Coralville, Iowa) |

| **Mutant Primer Sequences (5’-3’) (Mutations are underlined):** | | |  |
| --- | --- | --- | --- |
| **hnRNPA1 Mutant** | **Forward Primer** | **Reverse Primer** | **Template for PCR** |
| Fragment 1 | CCGGAATTCATGGATTACAAGGACGACGACGATAAGTCTAAGTCAGAGTCTCCTAAAGAG | ACTGTCTAGATTATGGTTCCACAACTCTTCC | Flag-hnRNP A1-WT (pCI Vector) |
| Fragment 2 | ACTGGAATTCATGGACTACAAAGACGATGACGACAAGAAGAGAGCTGTCTCCAGAG | ACTGTCTAGATTAACCGAAACCACCTCCACG | Flag-hnRNP A1-WT (pCI Vector) |
| Fragment 3 | ACTGGAATTCATGGACTACAAAGACGATGACGACAAGGGGAATGACAACTTCGGTG | TGCTCTAGATTAAAATCTTCTGCCACTGCC | Flag-hnRNP A1-WT (pCI Vector) |
| Fragment 131 | ccggaattcatgGACTACAAAGACGATGACGACAAGattgaagtgattgaaatcatg | Same as Fragment 3 | Flag-hnRNP A1-WT (pCI Vector) |
| Fragment 161 | ccggaattcatgGACTACAAAGACGATGACGACAAGaagattgtcattcagaaatac | Same as Fragment 3 | Flag-hnRNP A1-WT (pCI Vector) |
| Fragment 193 | ccggaattcatgGACTACAAAGACGATGACGACAAGcaaagaggtcgaagtggttc | Same as Fragment 3 | Flag-hnRNP A1-WT (pCI Vector) |
| SASSS Mutant 1 | AGAGATGGCTGCTGCTGCAGCCAGCCAAAGAGGTCG | CGACCTCTTTGGCTGGCTGCAGCAGCAGCCATCTCT | Flag-hnRNP A1-WT (pCI Vector) |
| SASSS Mutant 2 | AGAGATGGCTGCTGCTGCAGCCGCCCAAAGAGGTCG | CGACCTCTTTCGGCGGCTGCAGCAGCAGCCATCTCT | Flag-hnRNP A1-WT (pCI Vector) |
| T169A | GAAATACCATgctGTGAATGGCC | TGAATGACAATCTTATCCACG | Flag-hnRNP A1-WT (pCI Vector) |
| S182A | AAAAGCCCTGgcaAAGCAAGAGA | CTAACTTCACAGTTGTGGCC | Flag-hnRNP A1-WT (pCI Vector) |
| S197A | AAGAGGTCGAgctGGTTCTGGAAACTTTGG | TGGCTGGATGAAGCACTA | Flag-hnRNP A1-WT (pCI Vector) |
| S199A | TCGAAGTGGTgctGGAAACTTTG | CCTCTTTGGCTGGATGAAG | Flag-hnRNP A1-WT (pCI Vector) |
| S197A  S199A | tgctGGAAACTTTGGTGGTGGTC | ccagcTCGACCTCTTTGGCTGGA | Flag-hnRNP A1-WT (pCI Vector) |
| T169A  S197A  S199A | Same as T169A Forward and Reverse Primers | | Flag-hnRNP A1-S197AS199A (pCI Vector) |
| S182A  S197A  S199A | Same as S182A Forward and Reverse Primers | | Flag-hnRNP A1-S197AS199A (pCI Vector) |
| T169A  S182A  S197A  S199A | Same as S182A Forward and Reverse Primers | | Flag-hnRNP A1-T169AS197AS199A (pCI Vector) |
| S252A | GGATTTGGTAATGATGGAGCCAATTTTGGAGGTGG | CCACCACCTCCAAAATTGGCTCCATCATTACCAAATCC | Flag-hnRNP A1-WT (pCI Vector) |
| S259A | AATTTTGGAGGTGGTGGAGCCTACAATGATTTTGGG | CCCAAAATCATTGTAGGCTCCACCACCTCCAAAATT | Flag-hnRNP A1-WT (pCI Vector) |
| S270/271A | GGGAATTACAACAATCAGGCTGCAAATTTTGGACCCATG | CATGGGTCCAAAATTTGCAGCCTGATTGTTGTAATTCCC | Flag-hnRNP A1-WT (pCI Vector) |
| S285/286A | GGAAATTTTGGAGGCAGAGCCGCTGGCCCCTATGGCGG | CCGCCATAGGGGCCAGCGGCTCTGCCTCCAAAATTTCC | Flag-hnRNP A1-WT (pCI Vector) |
| **ARK5-Kinase Dead (KD) mutant: K84A point mutant** | | | |
| ARK5-KD | GGTTGCTATAGCATCCATTCGTAAGGAC | ACTCGGCCAGAAAACCTC | GST-ARK5 (pDEST27 vector) |

| **Western Blot and Immunofluorescence Antibodies:** | | | | |
| --- | --- | --- | --- | --- |
| **Antibodies** | | **Source** | **Cat. # (Company)** | **Application** |
| Primary | ARK5 | Rabbit | 4458 (Cell Signaling, NEB Ltd., Whitby, ON) | WB, IF |
|  | Bcl-xL | Rabbit | 2762 (Cell Signaling) | WB, IF |
|  | hnRNP A1 | Mouse | R9778 (Cell Signaling) | WB, IF |
|  | Flag | Mouse | 8146 (Cell Signaling) | WB |
|  | Tubulin | Mouse | Ab7291 (Abcam, Toronto, ON) | WB |
|  | Histone | Rabbit | 4499S (Cell Signaling) | WB |
| Secondary | Anti-rabbit IgG (HRP-linked) | Goat | 7074 (Cell Signaling) | WB |
|  | Anti-mouse IgG (HRP-linked) | Horse | 7076 (Cell Signaling) | WB |
|  | Alexa Fluor® 488 anti-mouse IgG | Goat | A11001 (Thermo Fisher Scientific, Ottawa, ON) | IF |
|  | Alexa Fluor® 680 anti-rabbit IgG | Goat | A21076 (Thermo Fisher Scientific) | IF |

| **Western Blot and Immunofluorescence Reagents:** | | |
| --- | --- | --- |
| **Reagents** | **Composition** | **Application** |
| Blocking | 5% Skim Milk in TBS-Tween | WB |
| Primary antibodies dilution | 1:1000 in 5% BSA in TBS-Tween | WB |
| Secondary antibodies dilution | 1:1000 in 5% Skim Milk in TBS-Tween | WB |
| Primary antibodies dilution | 1:300 in Triton X-100/BSA (hnRNPA1 & ARK5);  1:1000 in Triton X-100/BSA (Flag) | IF |
| Secondary antibodies | 1:1000 in Triton X-100/BSA | IF |
